# Profiling bacterial communities by MinION sequencing of ribosomal operons

**DOI:** 10.1186/s40168-017-0336-9

**Published:** 2017-09-15

**Authors:** Lee J. Kerkhof, Kevin P. Dillon, Max M. Häggblom, Lora R. McGuinness

**Affiliations:** 1 Department of Marine and Coastal Sciences, Rutgers University, 71 Dudley Rd, New Brunswick, NJ 08901-8521 USA; 2 Department of Environmental Sciences, Rutgers University, New Brunswick, NJ 08901 USA; 3 Department of Biochemistry and Microbiology, Rutgers University, New Brunswick, NJ 08901 USA

**Keywords:** Microbiota, Ribosomal operon, MinION, Species/strain-level resolution

## Abstract

**Background:**

An approach utilizing the long-read capability of the Oxford Nanopore MinION to rapidly sequence bacterial ribosomal operons of complex natural communities was developed. Microbial fingerprinting employs domain-specific forward primers (16S rRNA subunit), reverse primers (23S rRNA subunit), and a high-fidelity Taq polymerase with proofreading capabilities. Amplicons contained both ribosomal subunits for broad-based phylogenetic assignment (~ 3900 bp of sequence), plus the intergenic spacer (ITS) region (~ 300 bp) for potential strain-specific identification.

**Results:**

To test the approach, bacterial rRNA operons (~ 4200 bp) were amplified from six DNA samples employing a mixture of farm soil and bioreactor DNA in known concentrations. Each DNA sample mixture was barcoded, sequenced in quadruplicate (*n* = 24), on two separate 6-h runs using the MinION system (R7.3 flow cell; MAP005 and 006 chemistry). From nearly 90,000 MinION reads, roughly 33,000 forward and reverse sequences were obtained. This yielded over 10,000 2D sequences which were analyzed using a simplified data analysis pipeline based on NCBI Blast and assembly with Geneious software. The method could detect over 1000 operational taxonomic units in the sample sets in a quantitative manner. Global sequence coverage for the various rRNA operons ranged from 1 to 1951x. An iterative assembly scheme was developed to reconstruct those rRNA operons with > 35x coverage from a set of 30 operational taxonomic units (OTUs) among the *Proteobacteria*, *Actinobacteria*, *Acidobacteria*, *Firmicutes*, and *Gemmatimonadetes*. Phylogenetic analysis of the 16S rRNA and 23S rRNA genes from each operon demonstrated similar tree topologies with species/strain-level resolution.

**Conclusions:**

This sequencing method represents a cost-effective way to profile microbial communities. Because the MinION is small, portable, and runs on a laptop, the possibility of microbiota characterization in the field or on robotic platforms becomes realistic.

**Electronic supplementary material:**

The online version of this article (10.1186/s40168-017-0336-9) contains supplementary material, which is available to authorized users.

## Background

Molecular biological approaches for the genetic analysis of environmental samples have become the most widely accepted way to characterize microbial communities. Initially, a clone and sequence scheme was largely used to characterize 5S or 16S rRNA genes [1, 2]. Later, direct profiling methods such as denaturing gradient gel electrophoresis (DGGE), terminal restriction fragment length polymorphism (TRFLP), or single strand conformation polymorphism (SSCP) analysis were employed to characterize complex communities [3–5]. More recently, 16S rRNA gene sequence data is collected using a suite of “next generation” sequencing platforms (e.g., 454, Illumina, Ion Torrent) [6–9]. Although a large amount of data can be obtained in this manner, these recent approaches rely on expensive machines, bioinformatics training, and specialized computing facilities. In order to analyze the sequence data, a working knowledge of UNIX commands and Python scripts seems essential. Despite the computer software being freely available (e.g., QIIME and Mothur), the programs require the use of command lines and training in the proper UNIX syntax in order to function properly. Often the installation scripts and software dependencies become outdated quickly, and it is not always straightforward to install and/or operate. Furthermore, a server is generally needed to perform the analyses. Each of these requirements can place a significant monetary burden and a steep learning curve onto laboratories hoping to characterize bacterial communities. Finally, most second-generation sequencing platforms provide relatively short read lengths (200–400 bp) which limit the phylogenetic depth that can be achieved (with the exception of the PacBio system).

As an alternative approach, we tested if the portable DNA sequencer (MinION) from Oxford Nanopore Technologies (ONT) could be used to profile the microbiota using tools that can be purchased for a low cost and data analysis methods that are readily available to many laboratories. The MinION is a third-generation platform for direct sequencing of individual strands of DNA translocating nanoscale pores in a semiconductor membrane [10, 11]. A major advantage of the MinION is that it currently costs ~ $1000, connects to a laptop, collects/analyzes data in real time, and does not require specialized computer equipment or training for data analysis. For MinION sequencing, each DNA molecule has an adaptor ligated to one end, which interacts with a docking protein and binds to a nanopore. This docking protein regulates the speed by which the DNA traverses the membrane. The other end of the DNA fragment is ligated to a hairpin structure, which allows for the complementary strand to be sequenced as it flows through the pore. The DNA sequence is determined from 5-bp segments (k-mers) by measuring the change in electrical conductivity across the membrane as the DNA strand flows along the nanopore channel using hidden Markov models and Metrichor base calling software which is available to MinION users on the web. This approach generates 2D (double stranded; template plus complement) reads for single DNA molecules possessing both adaptor and hairpin, while 1D (single stranded; template or complement) reads are generated for DNA molecules possessing only the adaptor. (Those DNA molecules without adaptor or hairpin are removed during library preparation and are not detected in the analysis.)

For this study, we tested whether the MinION could be used to rapidly sequence bacterial ribosomal operons from complex environmental samples. To validate the approach, we generated a mixture of complex genomic DNA from two different sources where a large number of unknown microorganisms exist rather than a simple mock community of a few model organisms. After rRNA operon sequencing, each individual read was assigned to an operational taxonomic unit (OTU) by screening against an NCBI 16S rRNA gene database. An rRNA consensus sequence was then reconstructed for a particular OTU using an iterative alignment approach with a commercially available DNA software program (Geneious; < $900 per academic license) which can be run on Windows, Mac, or Linux operating systems. These efforts were designed to test if consensus building would yield data for environmental rRNA operons that are reproducible, quantitative, and similar to known rRNA genes within online databases.

**Fig. 1:**
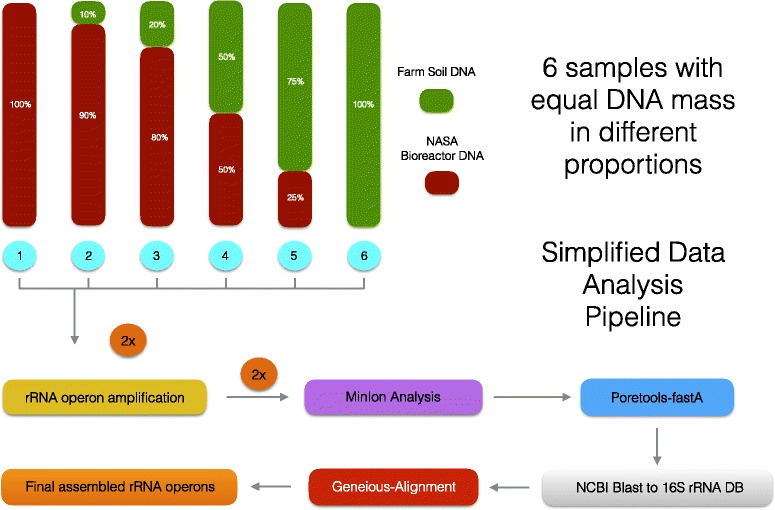
Schematic of the experimental design to test the MinION’s ability to resolve environmental rRNA operons in a quantitative manner indicating the simplified data analysis pipeline

In order to determine if the MinION can provide relative abundance data for the rRNA operons from environmental samples, genomic DNA from two different complex microbial communities were mixed in known concentrations (Rutgers farm soil and a NASA gray water bioreactor; [12]). These samples were chosen to represent environmental and engineered systems containing hundreds to thousands of different bacterial OTUs at varying concentrations. Six samples were constructed using mixtures of farm soil and bioreactor DNA at known concentrations to allow for replication and to test for the ability to provide relative abundance information (Fig. 1). Microbial fingerprinting employed domain-specific forward primers (16S rRNA gene subunit), domain-specific reverse primers (23S rRNA gene subunit), and a high-fidelity Taq polymerase with proofreading capabilities. The MinION data was processed with a user-friendly analysis pipeline in Geneious 10 that mirrors QIIME including denoising, OTU calling, assembling, and phylogenetic analysis (Additional file 1: Figure S1). Over 1000 OTUs (unique matches to the NCBI 16S rRNA gene database) were detected in the environmental dataset. Once the individual operon sequences were grouped by OTU, the various sequencing errors (i.e., miscalls, insertions, deletions; Additional file 1: Figure S2) were eliminated by consensus building. This consensus building approach demonstrated that nearly complete rRNA operons could be reconstructed from the MinION environmental dataset, containing 16S and 23S rRNA genes from members of different bacterial phyla. Both ribosomal RNA subunits yielded near identical phylogenetic tree topologies. Furthermore, MinION rRNA operon profiling was shown to be quantitative and provide species/strain-level resolution. Therefore, the MinION is a cost-effective alternative for profiling the microbiota that is small and portable and can be used in the field or on autonomous platforms.

## Results

**Fig. 2:**
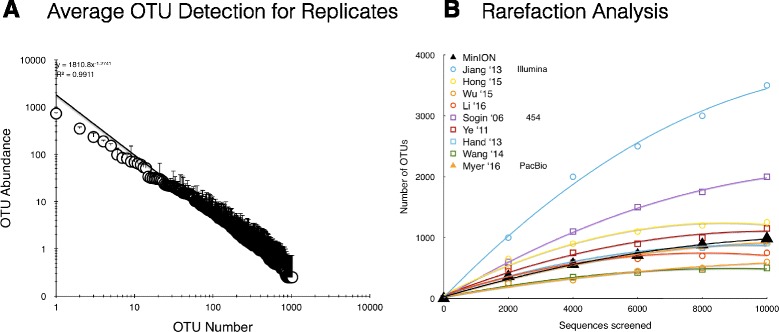
**a** Average frequency of operational taxonomic units (OTUs) by DMegaBlast screening of the 2D sequence reads. Error bars representing the standard deviation are in the positive direction only. **b** Rarefaction analysis of the MinION reads (closed black triangle) compared with published Illumina (open circles), 454 (open squares), and PacBio (closed orange triangle) studies as indicated for the symbol codes

Soil and bioreactor DNA were combined in known proportions to generate six sample communities to test the ability of the MinION to sequence nearly complete rRNA operons and determine if read numbers could measure the relative abundance of the different OTUs (Fig. 1). After purification of genomic DNA with Agencourt AMPure beads (Beckman Coulter; Brea, CA, USA), a 4.2-kb amplicon was created from the bacteria rRNA operons using 16S rRNA gene (27F) and 23S rRNA gene (2241R) primers (Additional file 1: Figure S3). The amplicons were barcoded using slight modifications of the Oxford Nanopore barcoding kit and R7.3 flow cells/chemistry. This yielded 12 barcoded samples per flow cell. A second set of rRNA operon libraries was also created for biological replication (e.g., separate DNA extractions, amplification, barcoding, ligations) and sequenced on a second flow cell, yielding quadruplicate sample replication for this study. Overall, nearly 90,000 reads totaling over 350 Mbp were obtained in two 6-h runs on the MinION. Most of the reads were 1D, representing sequences predominantly in the forward or reverse direction (Additional file 1: Table S1). However, roughly 33,000 reads containing both forward and reverse strands were recovered, yielding over 10,000 2D sequences generated by Metrichor. The MinION reads were analyzed via a simplified data analysis pipeline based on a local Discontinuous MegaBLAST search to a 16S rRNA gene database (bacteria and archaea; Bioproject 33175) using Geneious 10.1.2. This BLAST approach could detect over 1000 OTUs within the global data set (Fig. 2a) with coverage ranging from 1 to 1951x (Table 1). To ascertain if MinION yielded comparable proportions of identified OTUs with respect to the number of raw sequence reads as other next-generation sequencing platforms, rarefaction analysis was performed (Fig. 2b). Our results were similar to other published reports of microbial diversity using Illumina, pyrosequencing, and PacBio methods from mangroves, soils, dhole/dog feces, aquaculture ponds, the deep sea, waste water bioreactors, fruit fly gut, and cow rumen (Illumina methods-[13–16]; 454 methods- [6, 17–19]; PacBio-[20]). However, MinION OTU yields for the bioreactor and soil DNA were lower than studies of the marine samples. Additionally, we screened the MinION reads using ARB/SINA and the Greengenes, RDP, and SILVA SSU databases. This re-analysis indicated that NCBI and SILVA databases provided the lowest percentage of unclassified OTUs (i.e., either defined as unclassified in the database or < 70% identity over 500 bp; Additional file 1: Figure S4).

**Table 1:** Discontinuous MegaBLAST results for the rRNA consensus operons

NCBI top hit	Phylum	Bacterium	x coverage	Size (bp)	16S % ID*	Overlap*
gi|270610434|gb|GU187030.1|	*Acidobacteria*	*Acidobacteria bacterium IGE-003*	91	4022	83.8 ± 3.1	1345 ± 161
gi|584432076|gb|KF840371.1|	*Acidobacteria*	*Stenotrophobacter terrae*	59	4269	82.6 ± 5.0	1445 ± 83
gi|270610434|gb|GU187030.1||	*Acidobacteria*	Acidobacteria bacterium IGE-003 ***	56	4088	90.0 ± 4.7	622 ± 251
gi|270610434|gb|GU187030.1|	*Acidobacteria*	Acidobacteria bacterium IGE-003 ***	37	3933	84.7 ± 2.7	1324 ± 128
gi|645321086|ref|NR_118138.1|	*Actinobacteria*	*Gaiella occulata*	444	4006	86.6 ± 2.6	1483 ± 69
gi|283945692|gb|CP001854.1|	*Actinobacteria*	*Conexibacter woesei*	161	4148	88.3 ± 3.7	1472 ± 85
gi|631251774|ref|NR_112972.1|	*Actinobacteria*	*Aciditerrimonas ferrireducens*	98	3803	87.6 ± 1.4	1497 ± 33
gi|772393478|gb|KP174569.1	*Actinobacteria*	*Iamia majanohamensis*	50	4257	87.2 ± 1.6	1509 ± 41
gi|566084828|ref|NR_108192.1|	*Actinobacteria*	*Solirubrobacter ginsenosidimutans*	45	3655	86.1 ± 3.5	1446 ± 88
gi|343200946|ref|NR_041633.1|	*Actinobacteria*	*Ilumatobacter fluminis*	41	4167	89.3 ± 1.9	1279 ± 38
gi|294768454|gb|GU905013.1|	*Firmicutes*	*Enterococcus sp. CSL 7544–3*	68	4167	96.7 ± 2.1	1491 ± 52
gi|219846897|ref|NR_026489.1|	*Firmicutes*	*Vagococcus fluvialis*	46	4153	97.0 ± 2.1	1489 ± 51
gi|37961728|gb|AY234571.1|	*Gemmatimonadetes*	*Bacterium Ellin5220*	316	3611	84.0 ± 2.7	1039 ± 6
gi|68146509|emb|AJ867290.1|	*Proteobacteria-alpha*	*Ochrobactrum anthropi*	61	4567	98.2 ± 0.8	1530 ± 24
gi|219878237|ref|NR_025376.1	*Proteobacteria-beta*	*Comomonas nitrativorans*	941	4623	94.4 ± 1.0	1496 ± 40
gi|111378460|gb|DQ836252.1|	*Proteobacteria-beta*	*Comomonas denitrificans*	384	4585	96.3 ± 1.4	1501 ± 43
gi|698376506|gb|KM210263.1|	*Proteobacteria-beta*	*Castellaniella sp. ADC-27*	118	4652	95.2 ± 1.5	1502 ± 51
gi|589264544|emb|HG916765.1|	*Proteobacteria-beta*	*Castellaniella defragrans*	88	4519	95.1 ± 1.4	1513 ± 43
gi|343201701|ref|NR_042427.1|	*Proteobacteria-beta*	*Acidovorax caeni*	80	4432	95.4 ± 0.8	1521 ± 30
gi|937501645|gb|KR136349.1|	*Proteobacteria-beta*	*Achromobacter xylosoxidans*	70	4093	97.6 ± 0.2	1521 ± 16
gi|219878311|ref|NR_025450.1|	*Proteobacteria-beta*	*Bacterium Ellin6067*	39	3967	85.5 ± 0.4	1551 ± 10
gi|410994849|gb|CP003920.1|	*Proteobacteria-epsilon*	*Sulfuricurvum kujiense*	148	4453	89.1 ± 3.7	1499 ± 45
gi|54887524|emb|AJ786786.1|	*Proteobacteria-gamma*	*Xanthomonas sp. R-20819*	1951	4479	95.1 ± 1.0	1521 ± 27
gi|28170769|dbj|AB101447.1|	*Proteobacteria-gamma*	*Xanthomonas axonopodis*	352	4471	94.7 ± 1.0	1531 ± 27
gi|828983113|gb|CP011657.1|	*Proteobacteria-gamma*	*Citrobacter freundii CAV1741*	244	4248	98.2 ± 0.3	1534 ± 11
gi|938169631|gb|CP012900.1|	*Proteobacteria-gamma*	*Stenotrophomonas acidaminiphila*	161	4272	92.5 ± 0.5	1253 ± 5
gi|28170769|dbj|AB101447.1|	*Proteobacteria-gamma*	Xanthomonas axonopodis ***	113	4367	94.2 ± 1.2	1457 ± 30
gi|927043620|gb|CP012554.1|	*Proteobacteria-gamma*	*Citrobacter freundii P10159*	80	4177	97.9 ± 0.4	1536 ± 14
gi|927043620|gb|CP012554.1|	*Proteobacteria-gamma*	Citrobacter freundii P10159 ***	55	4286	97.2 ± 0.3	1540 ± 11
gi|636559888|ref|NR_115948.1|	*Proteobacteria-gamma*	*Lysobacter dokdonensis*	38	4361	93.7 ± 0.5	1545 ± 16

*Averages ± stdev from the top 100 BLAST hits from the NR database; *** 16S rRNA genes with different top Discontinuous MegaBLAST hits using the 16S rRNA gene and NR databases

Because the 2D sequencing error rate for MinION reads has been reported at 12% [21], we performed a sensitivity analysis to determine whether MinION reads with comparable errors could be accurately assigned to an OTU by Discontinuous MegaBLAST. Three 16S rRNA gene sequences from the NCBI database (*Stenotrophomonas maltophilia*, *Comomonas nitrativorans*, *Comomonas denitrificans*) had random errors and indels introduced along the entire length creating copies with similarities ranging from 79 to 100% (Additional file 1: Figure S5). All these test sequences were screened by Discontinuous MegaBLAST as described above and were assigned to the proper source OTU with the appropriate substitution rate (Additional file 1: Table S2).

**Fig. 3:**
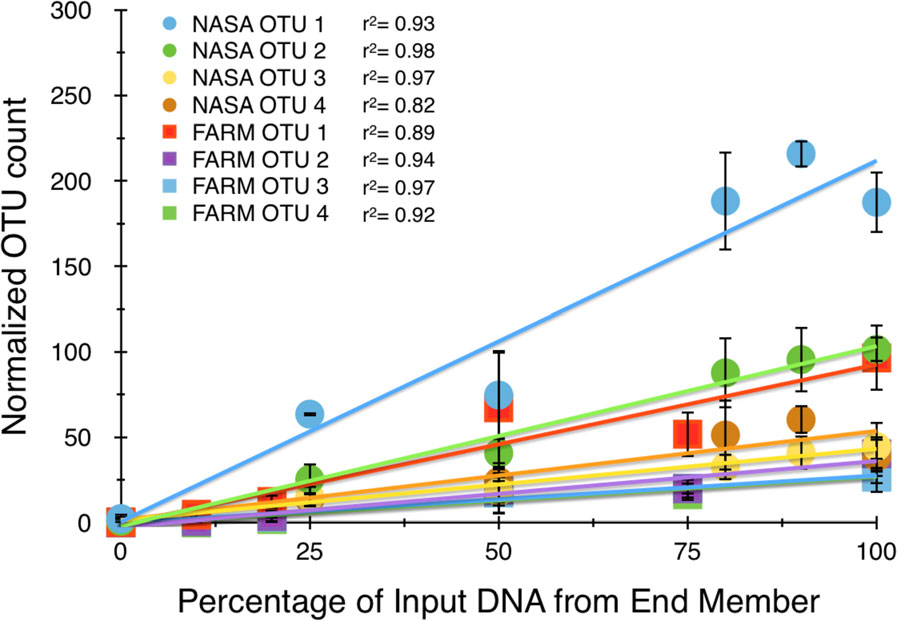
Quantitative response of the four most abundant OTU reads (farm soil and bioreactor; *y* axis) versus the percentage of input DNA from the end-member DNAs (*x* axis). Error bars represent the standard deviation

Although equal masses of amplicons from the various mixtures were used for library construction, the different barcodes did not provide a uniform number of 2D operon sequences per barcode (Additional file 1: Figure S6). However, once normalized, the 2D data indicated highly reproducible diversity patterns for the quadruplicate samples with most of the variability in OTUs below 10 hits (Fig. 2a). Since the contribution of the soil and bioreactor genomic DNA to each sample was known, it was possible to test if the various MinION OTUs were represented in a linear manner in the MinION reads. The top four normalized OTUs from the soil and bioreactor DNA provided a linear, quantitative signal with *r* ^2^ values ~ 0.9 for the most abundant OTUs (Fig. 3). Those less abundant OTUs with more than three data points for the DNA mixtures were also linearly correlated with the proportion of input DNA (*n* = 104). However, the robustness of this correlation declines with the number of OTUs < 10 within the sample set (Additional file 1: Figure S7).

**Fig. 4:**
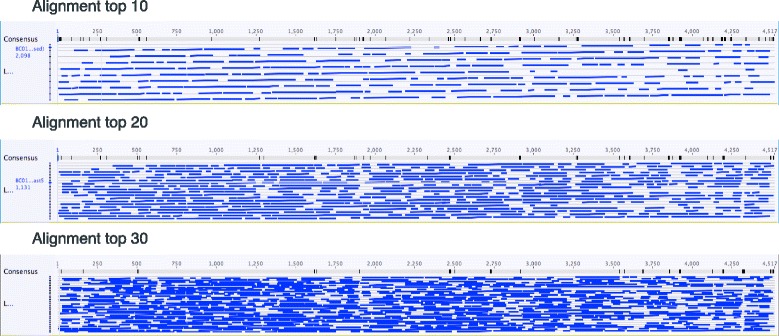
Example of iterative consensus building of rRNA operons using LastZ methods. The number of sequences used to build the consensus, and the disagreements (black bars) within the consensus are indicated

In order to reconstruct those rRNA operons with > 35x coverage, an iterative assembly scheme using LastZ alignment [22] was employed (Fig. 4). The results yielded nearly intact rRNA operons from members of the *Proteobacteria*, *Actinobacteria*, *Acidobacteria*, *Firmicutes*, and *Gemmatimonadetes*, containing most of the 16S and 23S rRNA subunits (examples in Additional file 1: Figure S8). The robustness and error rate of the consensus building scheme was tested by building consensus sequences from the four biological replicates. Here, three OTUs were identified from the bioreactor DNA end-member sample that had 30+ reads in each of the four barcoded samples (*Acidovorax wautersii*, *Comomonas nitrativorans*, *Stenotrophomonas rhizophilia*). The 16S rRNA genes from these biological replicates were independently aligned and used to build independent consensus sequences for each replicate as was done for the entire rRNA operon. All four replicate consensus sequences for the three OTUs were found to be identical (Additional file 1: Figure S9), indicating that our consensus building approach introduces minimal errors.

**Fig. 5:**
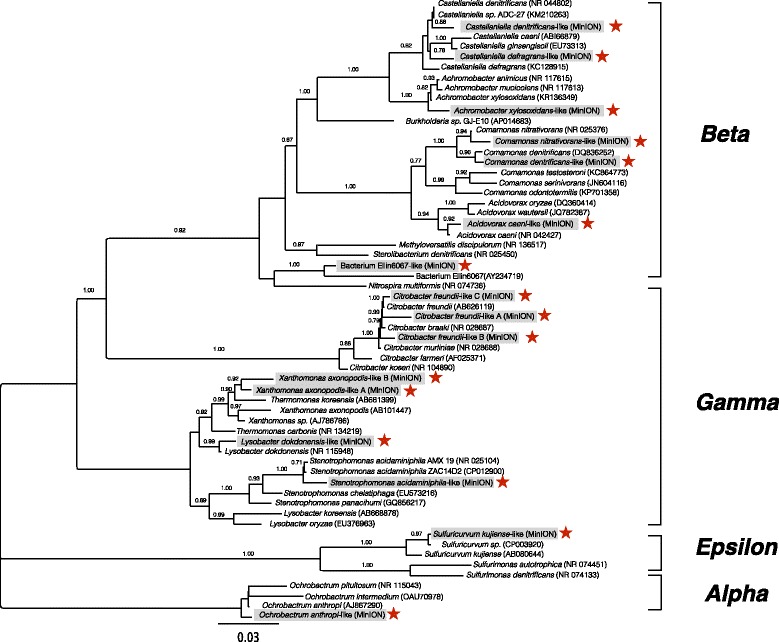
Phylogenetic tree reconstruction for the *Proteobacteria* 16S rRNA genes from farm soil, a gray water bioreactor, and closely related matching reference sequences using FastTree for 1292 unambiguously aligned bases. The MinION sequences are indicated by boxes and stars

**Fig. 6:**
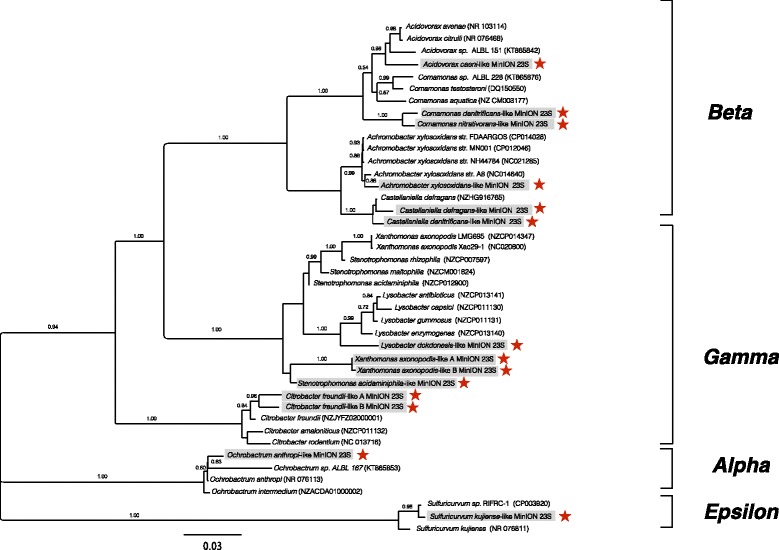
Phylogenetic tree reconstruction for the *Proteobacteria* 23S rRNA genes from farm soil, a gray water bioreactor, and closely related matching reference sequences using FastTree for 1767 unambiguously aligned bases. The MinION sequences are indicated by boxes and stars

To test whether the reconstructed rRNA operons contained chimeric rRNA genes, phylogenetic analysis was performed separately on the 16S and the 23S rRNA genes using 1292 and 1767 unambiguously aligned positions, respectively. Unfortunately, the 23S rRNA gene database is significantly smaller than the 16S rRNA gene database and UCHIME or similar software do not have a database that links the 16S and 23S rRNA genes. All MinION sequences using this method contained near identical phylogenetic tree topologies for both rRNA subunits as demonstrated for the *Proteobacteria* (Figs. 5 and 6; and the other bacterial phyla; Additional file 1: Figures S10-S13). Finally, the reconstructed 16S rRNA genes were re-screened against the NCBI NR database to assess if the top BLAST hits from the initial 16S rRNA database screen were also obtained from a much larger data set. In 26 out of 30 rRNA operons, the NR screen retrieved the identical top hit as the screen 16S rRNA dataset. In the remaining four rRNA operons, the 16S rRNA top hit was within the top three hits in the NR dataset. To gain a sense of the similarity between the reconstructed 16S rRNA genes and entries in the NR database, the average overlap and identity for the top 100 hits were calculated (Table 1). Most of the reconstructed rRNA operons (28 out of 30) retrieve nearly full-length BLAST hits from the NR database (> 1250 bp; most being ~ 1500 bp). Half of the reconstructed rRNA operons had identities > 93% for their respective matches. The other half of the reconstructed rRNA operons were not well represented in the 16S rRNA gene or NR databases and had similarities < 93%, as is often found when screening environmental samples.

## Discussion

Direct amplification of 16S rRNA genes from genomic DNA has revolutionized our understanding of the complexity of microbial communities. However, most recent efforts devoted to 16S rRNA gene discovery have focused more on the volume of sequences rather than the length of the sequence obtained from the molecule. It is now common to use high-throughput sequencing methods (e.g., Illumina, Ion Torrent, Pyrosequencing) to generate millions of short reads (often < 200–400 bp) and to report results at the phylum-order-family level. This approach inherently groups all members of a bacterial phyla-order-family together into a single unit and obscures species or strain-level dynamics that may be occurring in an environmental or experimental perturbation (e.g., light, temperature, nutrient addition). In this study, we tested a portable sequencing technology for the ability to distinguish bacterial species or strains in environmental samples. The Oxford MinION sequences single DNA molecules and enables very long reads to be obtained, compared to most second-generation sequencing approaches (> 10,000 bp vs 200 bp). When applied to rRNA gene characterization, this approach can provide nearly full-length rRNA operon sequence data yielding robust species resolution as demonstrated by both the tree topologies and the bootstrap values in Figs. 5 and 6. A comparable phylogenetic analysis using only the V4 and V5 regions of the 16S rRNA gene (~ 400 bp) did not provide robust species node resolution (Additional file 1: Figures S14–S15).

Although MinION sequencing of individual DNA molecules represents a major advance in characterizing entire operons and does not require in silico assembly, it should be noted that the Nanopore R5-R7 error rates (~ 15%; [23]) are often higher than the error rates for other sequencing systems, such as PacBio (25–160x higher; [24]). To date, nanopore sequencing has mostly been used to re-sequence known genomes for testing the ability to provide long reads and improve error correction. For example, there are reports of complete genomes being assembled using only R7 Nanopore sequence data with accuracies of 99.5% for *Escherichia coli* K12 [25] and 99.8% for *Francisella* strains [24]. Additionally, a combination of nanopore reads recruiting Illumina short reads to create a synthetic consensus for assembly/alignment (NaS fragments up to 60 kb in length) has been described with 99.99% accuracy for *Acinetobacter baylyi* [26]. However, it is conceivable that the higher MinION error rate could overestimate the number of OTUs that are detected, as has been reported for the V3–V5 regions of the 16S rRNA genes using MiSeq approaches [27]. Using the shorter MiSeq reads, the OTUs in a model bacterial community were overestimated by a factor of 1.1–9.6x, depending on the variable region and chimera removal using UCHIME. It is possible that the large number of singleton OTUs detected by the MinION reflect a higher sequence error. Interestingly, analysis of our DMegaBLAST results from the singleton MinION reads (> 40% query coverage) indicated that the average identity was 81 ± 5% over 1170 ± 150 bp for the 16S rRNA gene (*n* = 2409). Based on the sensitivity analysis (Additional file 1: Figure S5), the singleton DMegaBLAST results suggest that many of our rare reads are potentially being correctly assigned to an OTU. Furthermore, ONT has recently released R9 flow cells and chemistry for the MinION with improved throughput and lower error rates which promise to enable more accurate OTU assignment and a much greater number of near complete rRNA operons to be assembled from other complex environments.

Finally, other researchers have begun using the MinION to determine near a full-length sequence of 16S rRNA gene amplicons. Most of these studies have also tested model communities to demonstrate proof of concept. Specifically, researchers have tested *E. coli* K12 [28], a 3-member bacterial system containing *Streptococcus* and *Parvimonas* [29], a 20-member model community using representative DNA from different bacterial phyla (e.g., *Proteobacteria*, *Firmicutes*, *Bacteroides*, *Deinococcus*, *Actinobacteria*) [30, 31]. All studies found that the MinION could provide a nearly full-length sequence of 16S rRNA gene amplicons with accuracies ranging from 80 to 94% and could often obtain species-level resolution. Only a single study has utilized a complex “environmental”-type sample (mouse fecal material) [32] comparing Illumina and ONT Nanopore sequencing). While Shin et al. (2016) found nearly 1000 OTUs by Illumina methods, and they did not report the number of OTUs in their MinION data. However, they described the identification of more bacterial species using the nanopore data compared with the MiSeq (*n* = 16), and the authors could demonstrate robust phylogenetic resolution of species of *Bifidobacterium* and *Bacteroides*. In contrast with these prior studies, we used purified DNA from complex environment settings (soils and bioreactors), containing a large number of unknown bacterial species and grouped the various MinION sequences by OTU to remove sequencing errors using an iterative consensus building approach.

## Conclusions

Our analysis demonstrated that the MinION has the ability to provide rRNA operon sequence data of sufficient quality for characterizing the microbiota of complex environmental samples and provided results that are reproducible, quantitative, and consistent. Over 1000 OTUs could be detected from our test environmental sample mixture. However, further analysis of the errors in rare reads may be necessary to ensure accurate OTU assignment. The long-read capability of MinION allowed for robust bacterial species and strain resolution combining both 16S and 23S rRNA genes, consistent with previous reports [30, 32]. Additionally, improvements in chemistry and library prep have led to increasing accuracy from 66 to 92% within the last few years [33] and ONT has released a newer version of their analysis software (MinKnow v51.3) that allows for local base calling on the host computer, rather than in the cloud using Metrichor. Given the MinION’s low cost, small size, improving chemistry, and ability to analyze the nucleic acid data in real time, genetic analysis on mobile, and robotic platforms becomes feasible, as connectivity to the web is no longer required to analyze a sequence run.

## Methods

### DNA extraction

DNA from Rutgers farm soil and bioreactor samples [12] were extracted in triplicate (twice) using a modified CTAB extraction method [34]. Briefly, samples were amended with 100 μl of solution 1 (50 mM glucose, 10 mM EDTA, 25 mM Tris-Cl; pH 8.0) and subjected to five quick freeze/thaw cycles between liquid nitrogen temperatures and a 55 °C water bath. After these freeze/thaw cycles, 450 μL CTAB solution (4% CTAB, 100 mM Tris [pH 8.2], 20 mM EDTA, 1.4 M NaCl), 0.14 M β-mercaptoethanol was added. The samples were quickly extracted 2x with 800 μl phenol: chloroform: isoamyl alcohol; 25:24:1 (> pH 7.0). The aqueous phase of each extract was ethanol precipitated with the addition of 2 μg of glycogen. The triplicate extracts were combined into a single stock for the farm soil and bioreactor to create end-member DNAs of very different microbial communities for this study. These end-member DNAs were brought to the same concentration and combined in different ratios, respectively: 0/100, 10/90, 20/80, 50/50, 75/25, and 100/0 (farm soil DNA/bioreactor DNA) for MinION sequencing (Fig. 1). Further DNA purification was done by combining 20 μl of DNA, 20 μl of 6 M NaI, and 20 μl of Ampure beads (Beckman Coulter; Brea, CA, USA). After DNA binding on a vortexer mixer for 10 min, the beads were separated using a magnet and washed twice with freshly made 70% ethanol. DNA elution employed sterile water with a 55 °C treatment for 10 min.

### Amplification of rRNA operons

Bacterial ribosomal operons were amplified using modified 16S rRNA-27Forward primer (5′ TTT CTG TTG GTG CTG ATA TTG C-[barcode overhang for PCR labeling]-AGA GTT TGA TCC TGG CTC AG 3′) [35] and modified 23S rRNA-2241Reverse primer (5′ ACT TGC CTG TCG CTC TAT CTT C-[barcode overhang for PCR labeling]-ACC GCC CCA GTH AAA CT 3′) [36]. Ribosomal operon amplicons were generated using AMPure bead purified DNA as follows: 10 ng template DNA was combined with dNTP’s, five units of Universe High-Fidelity Hot Start DNA polymerase (Biomake LLC, Houston, TX, USA), primers, and PCR buffer. Amplification conditions were 5 min at 94 °C, followed by 27 cycles at 94 °C for 0.5 min and 72 °C for 1.5 min. At 18 cycles, 10 μl of amplification mixture was removed and stored at −80 °C. The amplification was allowed to proceed until 27 cycles and the product was visualized by agarose gel electrophoresis. Once clean, PCR product was observed, the 18 cycle mixture was cleaned with AMPure beads by bringing the volume up to 50 μl with water, adding 50 μl of 5 M NaCl, 50 μl of 30% PEG/1.5 M NaCl, and 7 μl of Ampure Beads. Ethanol washing and resuspension in 10 μl of water were done as described above. Purified DNA (1 μl) after 18 PCR cycles was added to a tube containing the ONT barcodes, and the amplification was repeated.

### Library preparation

Library construction for the MinION relies on ligation of adaptor and hairpin to rRNA amplicons in order to perform nanopore sequencing. For this study, 100 ng of each barcoded amplicon were combined (1200 ng total) into DNA Lo-Bind tubes at a volume of 85 μl (by adding reagent grade PCR water) with 10 μl end-repair buffer and 5 μl of the end-repair enzyme (New England Biolabs, Ipswich, MA, USA). After a 20 min incubation at room temperature, the end-repair reaction was concentrated/purified by adding 100 μl of 5 M NaCl, 100 μl of 30% PEG/1.5 M NaCl, and 15 μl of Ampure Beads and allowed to bind for 10 min on a vortex shaker. The beads were removed from the supernatant using a magnet and washed twice with freshly made 70% ethanol. The end-repaired DNA was eluted in 25 μl of water at 55 °C for 10 min and dA-tailing was done by adding 3 μl of tailing buffer and 2 μl of enzyme (NEB) and incubating at 37 °C for 10 min. The DNA was re-purified on AMPure beads using the NaCl/PEG protocol as above and re-suspended in 15 μl of water at 55 °C for 10 min.

For the ligation, “half-reactions” were utilized with slight modifications. Fifteen microliters of DNA was combined with 9 μl of water, 5 μl of ONT adaptor mix, 1 μl of HP adaptor, and 25 μl of Blunt/TA ligase master mix (NEB). Additionally, a critical modification was to add 1–2 μl of freshly prepared ATP solution (~ 4 mg/ml). The mixture was incubated for 10 min at room temperature, then 0.5 μl of HP tether was added, and the reaction was allowed to incubate an additional 10 min. The library was then purified using streptavidin C1 magnetic beads as per ONT instructions with the exception that the elution was done by incubating the bead in 25 μl elution buffer overnight at 4 °C then by a 30-min incubation at 37 °C. The library was loaded into R7 flow cells and run as per the manufacturer’s instructions.

### QA/QC on Geneious

After sequencing on the MinION, the 2D reads were opened using Poretools [37] and the corresponding fastA files were exported. These sequence files were subjected to QA/QC analysis by annotating in Geneious using six pairs each of universal 16S rRNA primer sequences (27F, 343F, 518F, 907F, 1392F, and 1492F) [35] and 23S rRNA primer sequences (129F, 473F, 820F, 1623F, 2069F, and 2758F) [35, 36]. Only those files between 4 and 5 kb and containing at least two rRNA priming sites were retained for further analysis (~ 85% of the 2D sequences). These files were oriented in a uniform direction, and the 16S rRNA sequences were extracted in Geneious (Additional file 1: Figure S16).

### OTU determination

The MinION 16S rRNA genes for each barcode were screened against an NCBI 16S rRNA gene bacterial and archaeal database (Bioproject 33175) using Discontinuous MegaBLAST in Geneious 10.1.2. Settings included a word size of 11, gap cost of 5/2, scoring of 2/−3, and a seed length of 18. The top BLAST output was exported as .csv files and opened in a spreadsheet program (e.g., Numbers, Excel) to group by best BLAST hit, count the number of OTUs, and parse for comparisons across samples. Additionally, the MinION sequences were analyzed by SINA online at the Arb-SILVA website (https://www.arb-silva.de/aligner). Settings included rejecting sequences < 70% identity, search-kmer candidates 100, lca-quorum 0.8, search-kmer length 10 using the SILVA, RDP, and Greengenes RefNR databases.

### Consensus reconstruction

Thirty sequences representing a single OTU with the same top DMegaBLAST scores were copied into a separate folder and used to build a consensus rRNA operon from the host organism via an iterative LastZ alignment approach [22]. Initially, ten sequences were selected to build a consensus sequence by MUSCLE alignment using Geneious. This consensus was exported as text and imported in Pages to remove gaps. This MUSCLE consensus was used to re-align the original 10 operon sequences into a new LastZ consensus (termed “con 1A”). “Con 1A” was then used to align 20 of the rRNA operon sequences with LastZ to create “con 2A”. “Con 2A” was then used to align 30 of the rRNA operons to create a final consensus. The process was repeated with the next set of operon sequences (e.g., con 1B) and with the final set of 10 sequences (con 1C, etc.) All three final consensus sequences (A, B, C) were assessed for coverage and sequence length to choose a final consensus that best represents the OTU (Fig. 4). This final rRNA consensus sequence was annotated by selecting regions excluding the priming sites and screened by BLAST against the NR database to determine the full extent of the 16S and 23S rRNA genes.

### Phylogenetic tree analysis

A maximum likelihood method (FastTree 2.1.5 with default settings) was used to reconstruct phylogenetic trees by first aligning full-length sequence for the ribosomal subunits with MUSCLE. The alignment was edited in Geneious to retain only unambiguously aligned bases (16S rRNA genes-1292 bp and 23S rRNA genes-1767 bp).

## Additional file

Additional file 1: Figure S1. Comparison of the QIIME data analysis pipeline with this study of MinION data. **Figure S2.** Example of sequencing errors from individual MinION reads. **Figure S3.** Schematic diagram indicating the PCR primer sites for rRNA operon amplification and an agarose gel demonstrating amplicons. **Figure S4.** Percent of OTU classification results for the MinION reads by database using Discontinuous MegaBLAST (NCBI) or the ARB/SINA website (https:www.arb-silva.de/aligner; Greengenes, RDP, and SILVA). **Figure S5.** Test sequences used for assessing Discontinuous MegaBLAST for assigning operational taxonomic units (OTUs) to individual MinION reads. **Figure S6**. The number of barcoded reads using equal masses of amplicons for library creation. **Figure S7.** Average linear correlation coefficients for less abundant OTUs (with 3 or more data points) not shown in Fig. 3. **Figure S8.** Examples of 10 re-constructed rRNA operons from the MinION. **Figure S9.** Comparison of 16S rRNA gene consensus sequences from reads in the 4 biological replicates. **Figure S10.** Phylogenetic tree re-construction for the Actinobacteria 16S rRNA genes. **Figure S11.** Phylogenetic tree re-construction for the Actinobacteria 23S rRNA genes. **Figure S12.** Phylogenetic tree re-construction for 16S rRNA genes from other bacterial phyla. **Figure S13.** Phylogenetic tree re-construction for 23S rRNA genes from other bacterial phyla. **Figure S14.** Phylogenetic tree re-construction for the Proteobacteria using FastTree for 400 unambiguously aligned bases of the V4-V5 regions from the 16S rRNA gene. **Figure S15.** Phylogenetic tree re-construction for the Proteobacteria using FastTree for 1292 unambiguously aligned bases from the nearly complete 16S rRNA gene. **Figure S16.** Example of QA/QC screening of the MinION raw data by Geneious. **Table S1.** MinION read statistics. **Table S2.** Results of screening the “mutated” copies of three 16S rRNA genes using Discontinuous MegaBLAST as described in the text. (PDF 3614 kb)
