# Supplementary material for: Profiling bacterial communities by MinION sequencing of ribosomal operons
Source: Microbiome. 2017 Sep 15;5:116. doi: 10.1186/s40168-017-0336-9 (PMC5599880; doi:10.1186/s40168-017-0336-9)
Supplement: Additional file 1: Figure S1. — Comparison of the QIIME data analysis pipeline with this study of MinION data. Figure S2. Example of sequencing errors from individual MinION reads. Figure S3. Schematic diagram indicating the PCR primer sites for rRNA operon amplification and an agarose gel demonstrating amplicons. Figure S4. Percent of OTU classification results for the MinION reads by database using Discontinuous MegaBLAST (NCBI) or the ARB/SINA website (https:www.arb-silva.de/aligner; Greengenes, RDP, and SILVA). Figure S5. Test sequences used for assessing Discontinuous MegaBLAST for assigning operational taxonomic units (OTUs) to individual MinION reads. Figure S6. The number of barcoded reads using equal masses of amplicons for library creation. Figure S7. Average linear correlation coefficients for less abundant OTUs (with 3 or more data points) not shown in Fig. 3. Figure S8. Examples of 10 re-constructed rRNA operons from the MinION. Figure S9. Comparison of 16S rRNA gene consensus sequences from reads in the 4 biological replicates. Figure S10. Phylogenetic tree re-construction for the Actinobacteria 16S rRNA genes. Figure S11. Phylogenetic tree re-construction for the Actinobacteria 23S rRNA genes. Figure S12. Phylogenetic tree re-construction for 16S rRNA genes from other bacterial phyla. Figure S13. Phylogenetic tree re-construction for 23S rRNA genes from other bacterial phyla. Figure S14. Phylogenetic tree re-construction for the Proteobacteria using FastTree for 400 unambiguously aligned bases of the V4-V5 regions from the 16S rRNA gene. Figure S15. Phylogenetic tree re-construction for the Proteobacteria using FastTree for 1292 unambiguously aligned bases from the nearly complete 16S rRNA gene. Figure S16. Example of QA/QC screening of the MinION raw data by Geneious. Table S1. MinION read statistics. Table S2. Results of screening the “mutated” copies of three 16S rRNA genes using Discontinuous MegaBLAST as described in the text. (PDF 3614 kb) [file 40168_2017_336_MOESM1_ESM.pdf]

Figure S1:

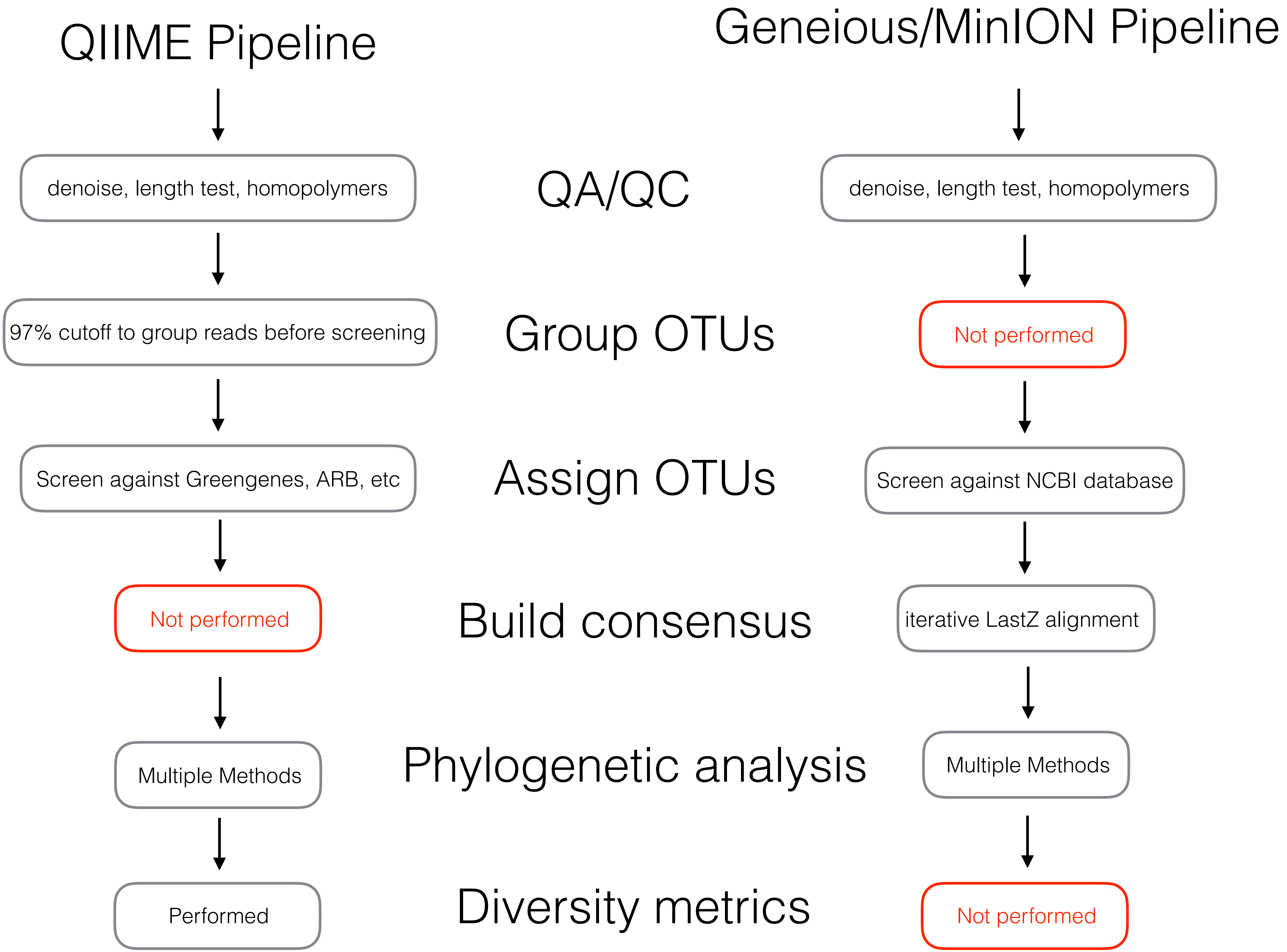
QIIME Pipeline
Geneious/MinION Pipeline
denoise, length test, homopolymers
QA/QC
denoise, length test, homopolymers
97% cutoff to group reads before screening
Group OTUs
Not performed
Screen against Greengenes, ARB, etc
Assign OTUs
Screen against NCBI database
Not performed
Build consensus
iterative LastZ alignment
Multiple Methods
Phylogenetic analysis
Multiple Methods
Performed
Diversity metrics
Not performed

Figure S2:

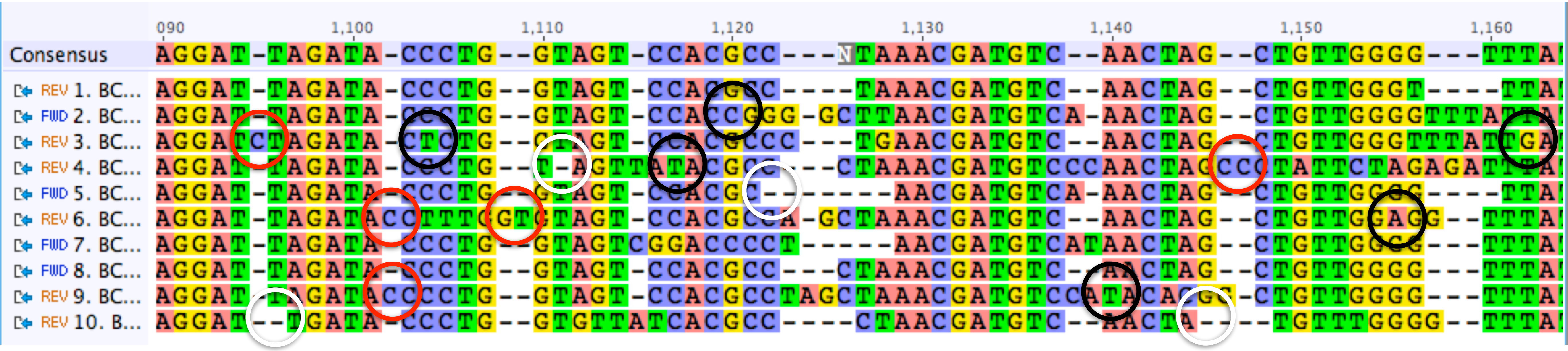


Insertions (red circle), deletions (white circle), and miscalled bases (black circle).

The consensus sequence is shown on the top row.

# Figure S3:

Schematic of a bacterial rRNA operon

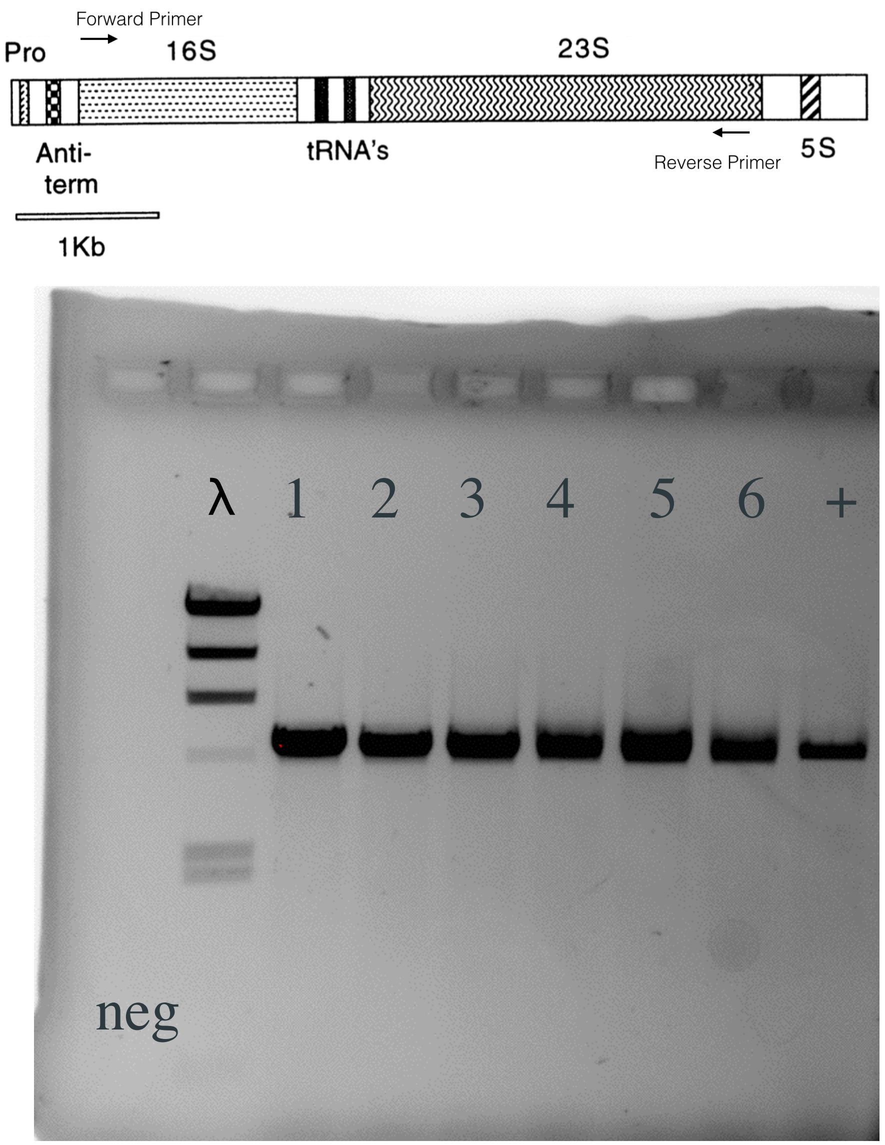


The lanes indicate the various barcoded samples, the PCR negative (neg) and positive (+) controls, and lambda molecular weight standard.

Figure S4:

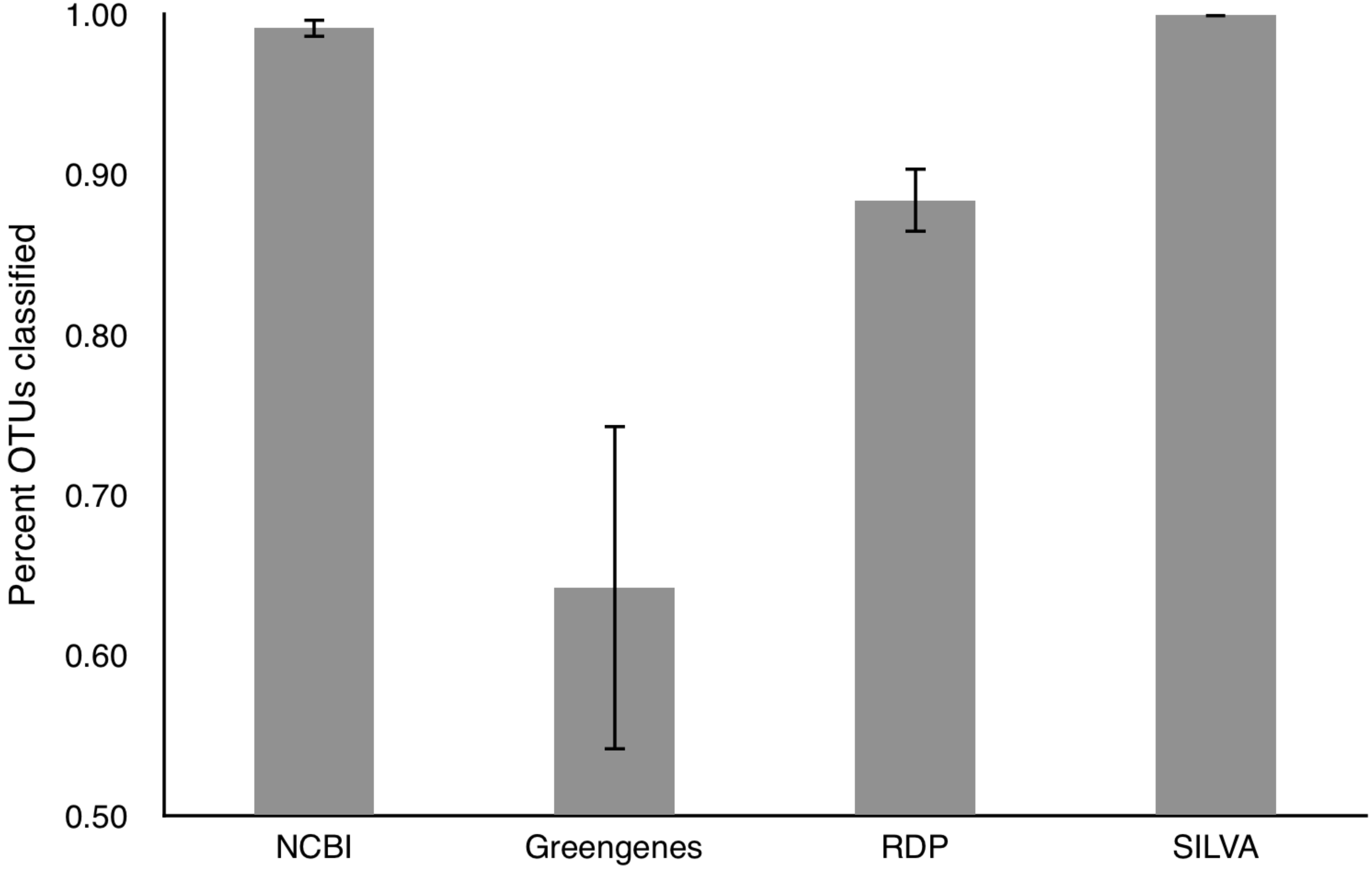


Unclassified OTUs are either defined as unclassified in the database or <70% identity over 500 bp. Data represents averages for the various biological replicates and the error bars are the standard deviation.

Figure S5:

*Stenotrophomonas maltophilia* (100-79% similarity)

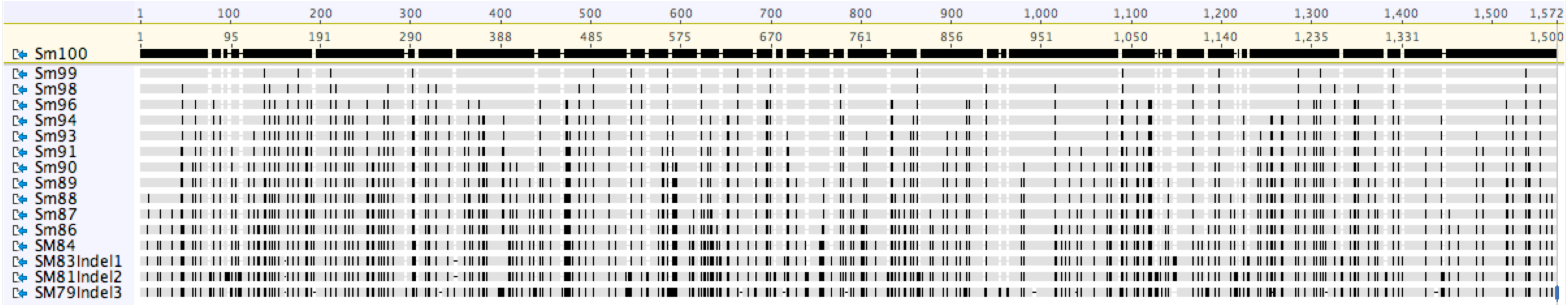


*Comomonas nitrativorans* (100-79% similarity)

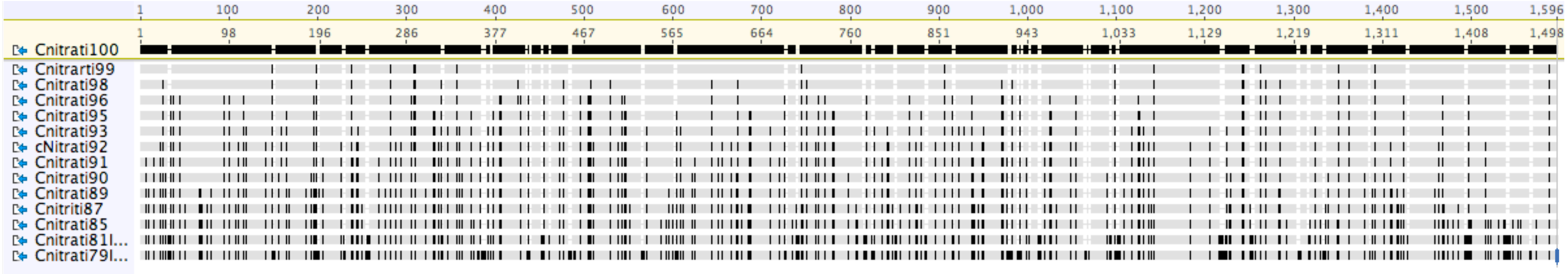


*Comomonas denitrificans* (100-79% similarity)

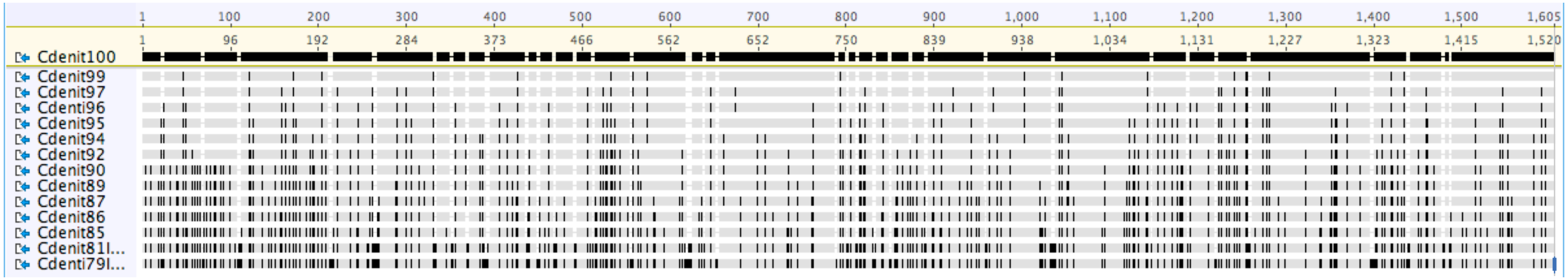


Random substitutions and insertions/deletions were created in three 16S rRNA genes to generate 12-13 sequences between 79-100% identity. The positions of the substitutions are indicated below by the black lines. All sequences were then screened in Geneious and the results are shown in Additional Table 2.

Figure S6:

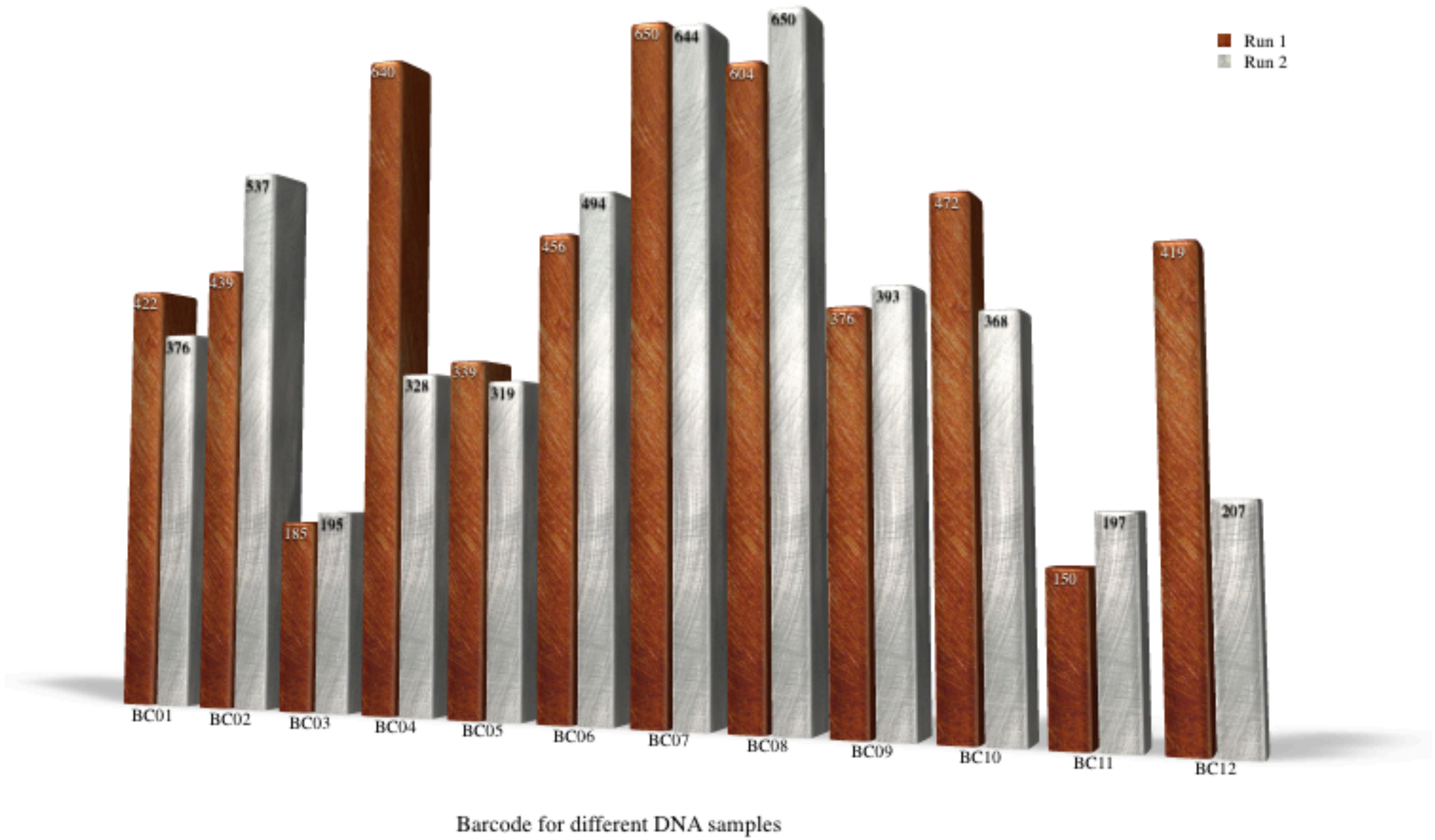
Run 1
Run 2
422
376
439
537
185
195
640
328
339
319
456
494
650
644
604
650
376
393
472
368
150
197
419
207
BC01
BC02
BC03
BC04
BC05
BC06
BC07
BC08
BC09
BC10
BC11
BC12
Barcode for different DNA samples

Figure S7:

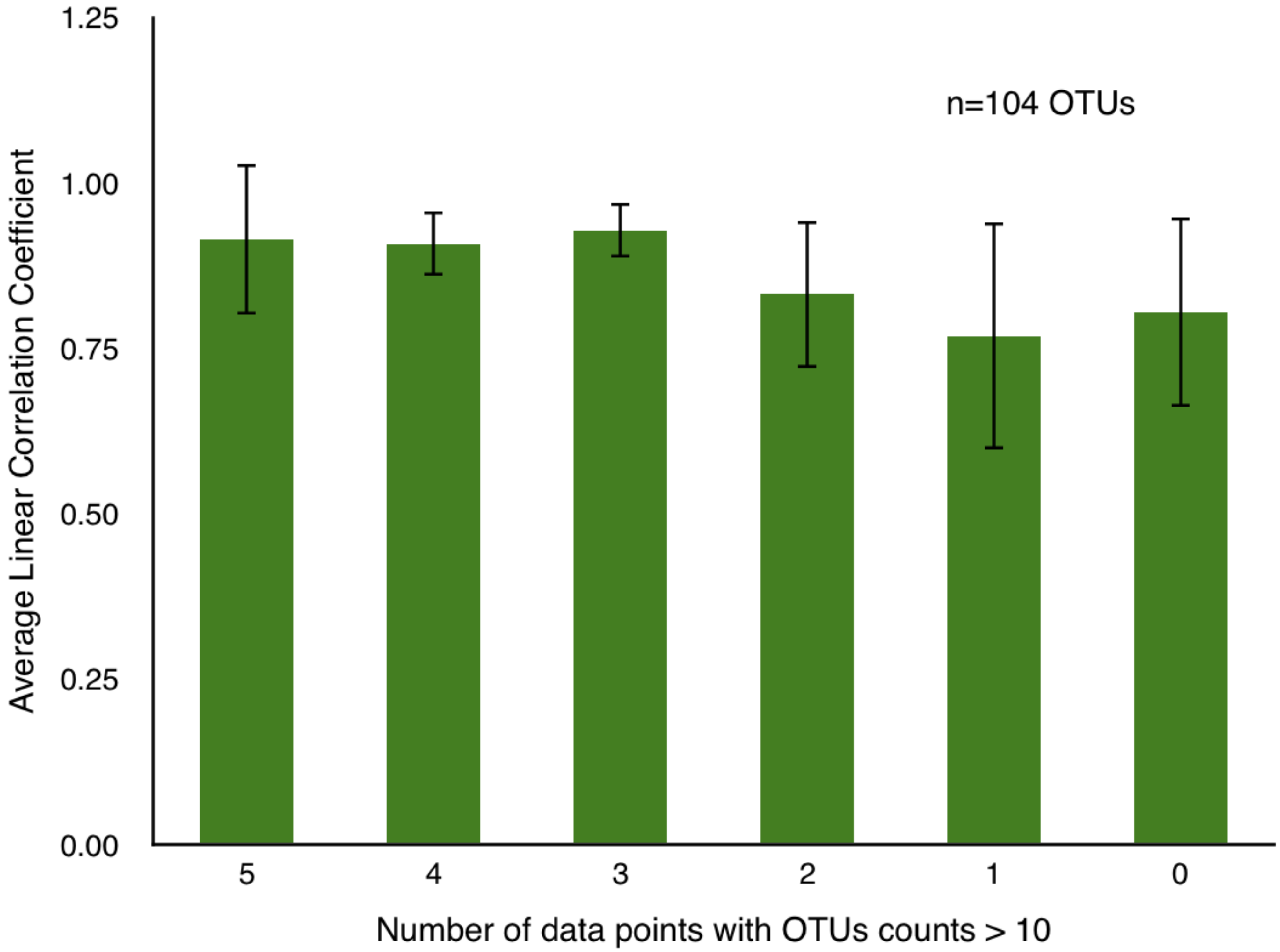


Samples are grouped into the number of data points with >10 OTUs. Error bars represent the standard deviation.

# Figure S8:

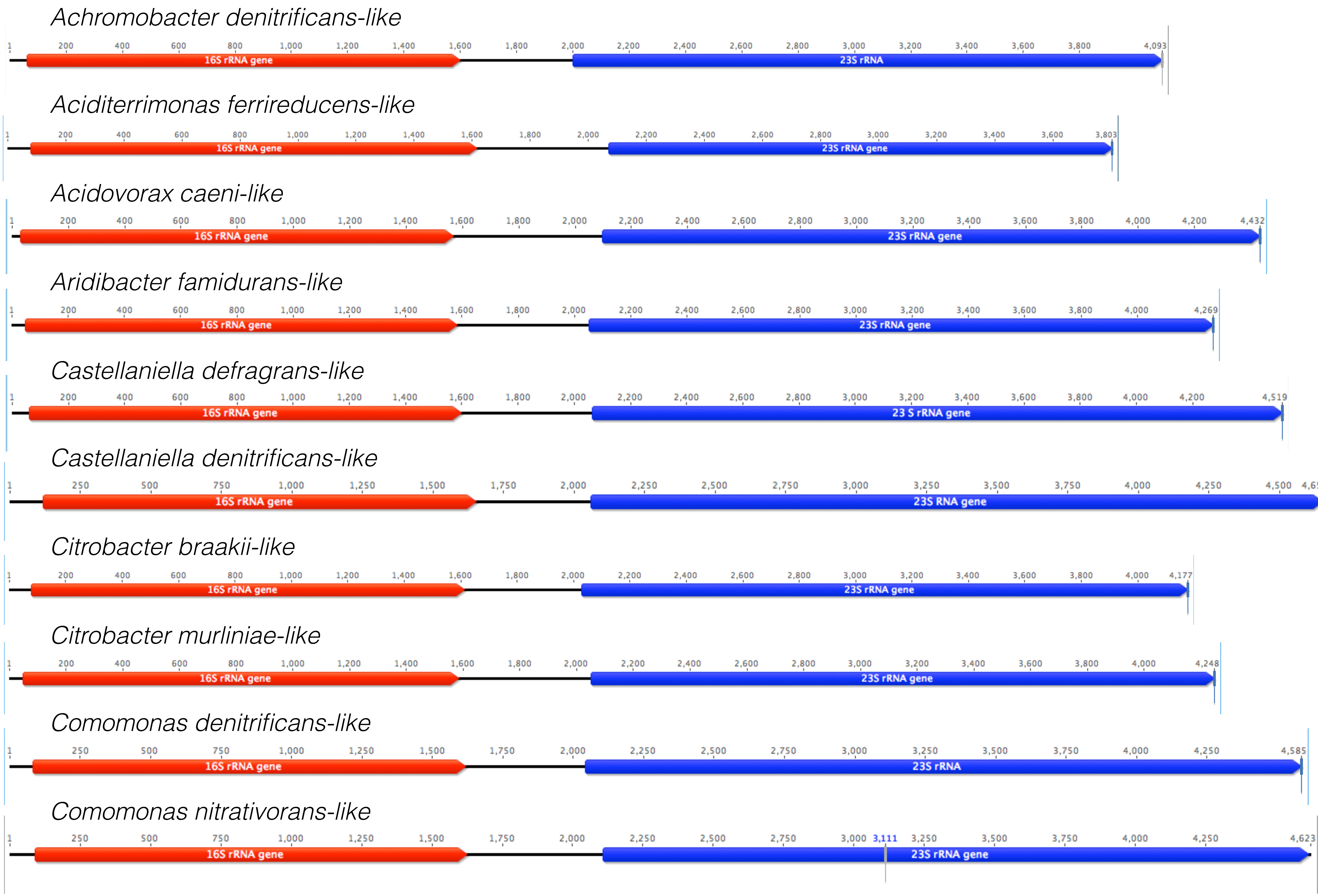


16S rRNA genes (red) and the 23S rRNA genes (blue) indicated.

# Figure S9:

*Acidovorax wautersii*

| | Biological Rep 1 | Biological Rep 2 | Biological Rep 3 | Biological Rep 4 |
|---|---|---|---|---|
| **Biological Rep 1** | - | 100 | 100 | 100 |
| **Biological Rep 2** | 100 | - | 100 | 100 |
| **Biological Rep 3** | 100 | 100 | - | 100 |
| **Biological Rep 4** | 100 | 100 | 100 | - |

*Comomonas nitrativorans*

| | Biological Rep 1 | Biological Rep 2 | Biological Rep 3 | Biological Rep 4 |
|---|---|---|---|---|
| **Biological Rep 1** | - | 100 | 100 | 100 |
| **Biological Rep 2** | 100 | - | 100 | 100 |
| **Biological Rep 3** | 100 | 100 | - | 100 |
| **Biological Rep 4** | 100 | 100 | 100 | - |

*Stenotrophomonas rhizophilia*

| | Biological Rep 1 | Biological Rep 2 | Biological Rep 3 | Biological Rep 4 |
|---|---|---|---|---|
| **Biological Rep 1** | - | 100 | 100 | 100 |
| **Biological Rep 2** | 100 | - | 100 | 100 |
| **Biological Rep 3** | 100 | 100 | - | 100 |
| **Biological Rep 4** | 100 | 100 | 100 | - |

DNA from each biological replicate was amplified, sequenced, and processed separately to assess errors from consensus building. All 16S rRNA gene consensus sequences from the replicates were found to be identical.

## Figure S10:

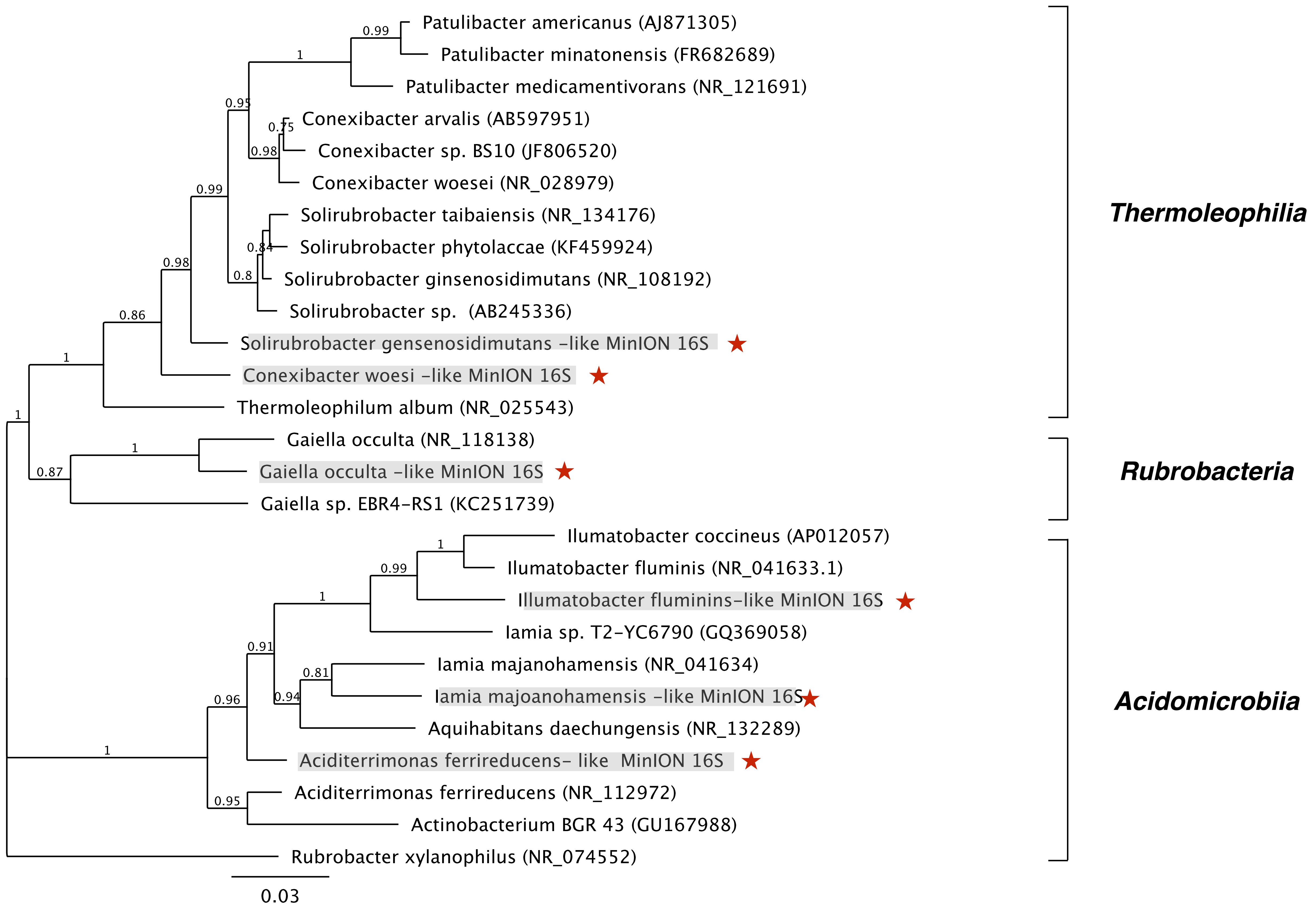


Tree reconstructions using FastTree for 1252 unambiguously aligned bases.

The MinION sequences are indicated by boxes and stars.

Figure S11:

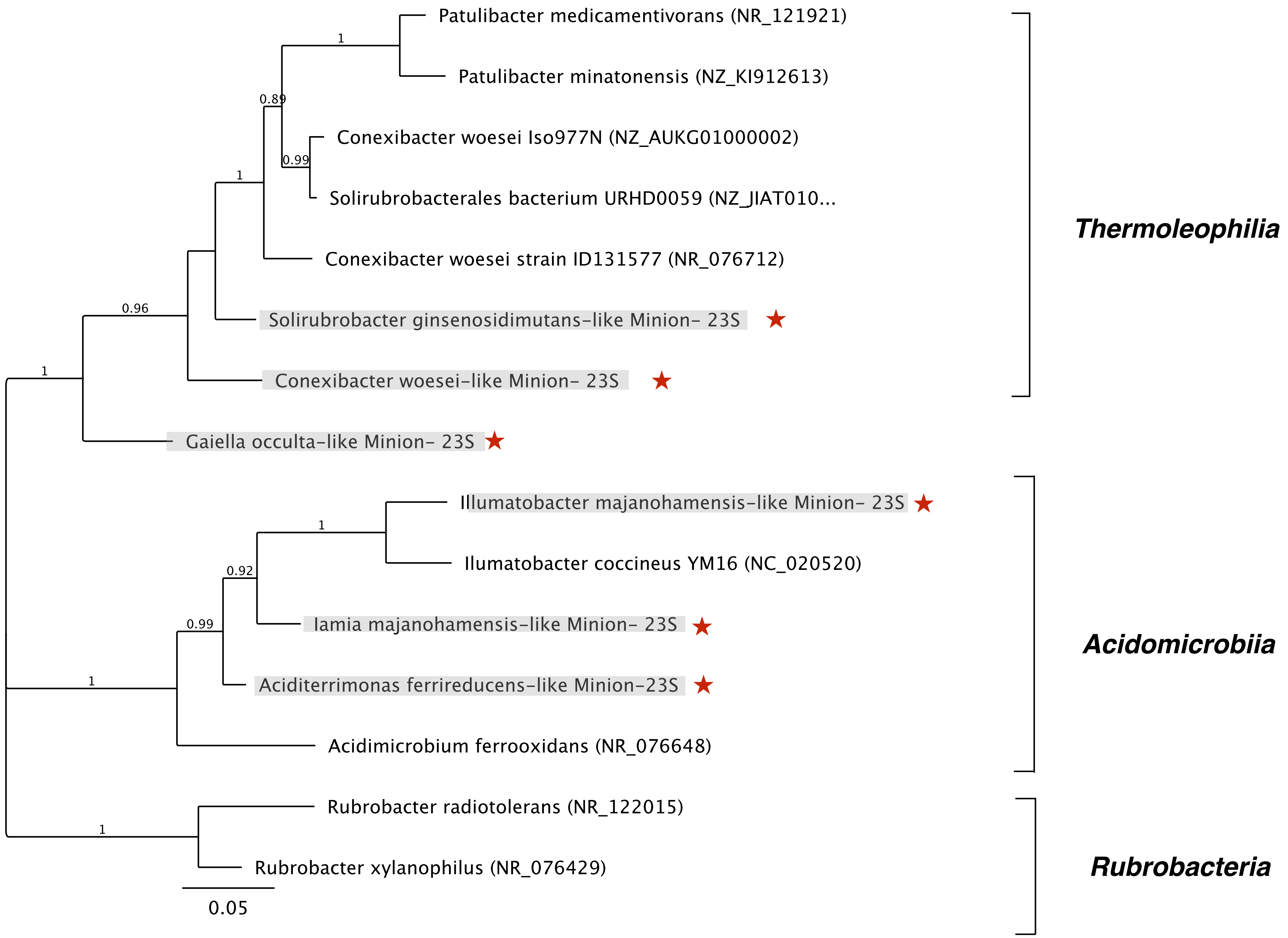


Tree reconstructions using FastTree for 1553 unambiguously aligned bases.

The MinION sequences are indicated by boxes and stars.

Figure S12:

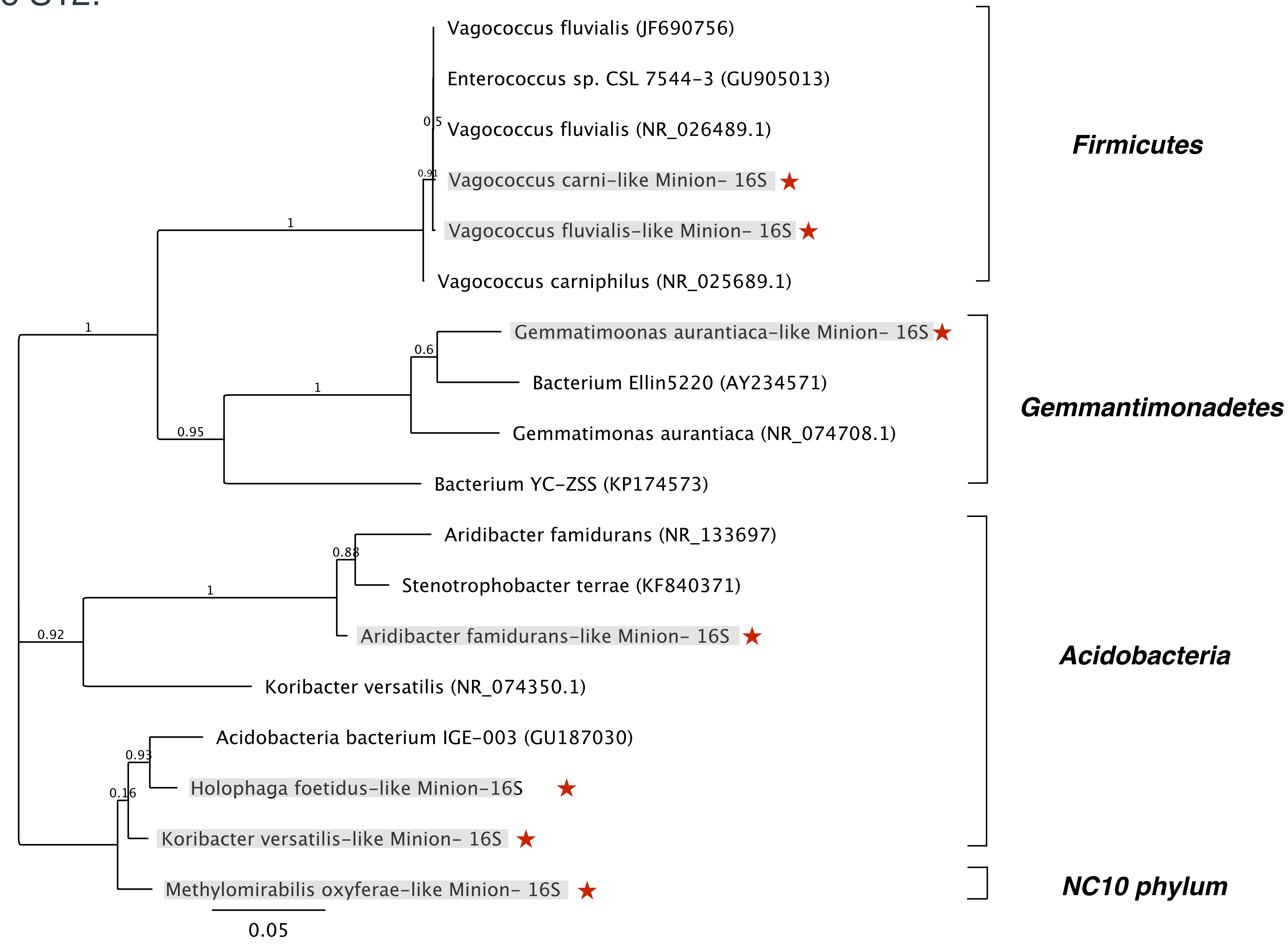


Tree reconstructions using FastTree for 1181 unambiguously aligned bases.

The MinION sequences are indicated by boxes and stars.

Figure S13:

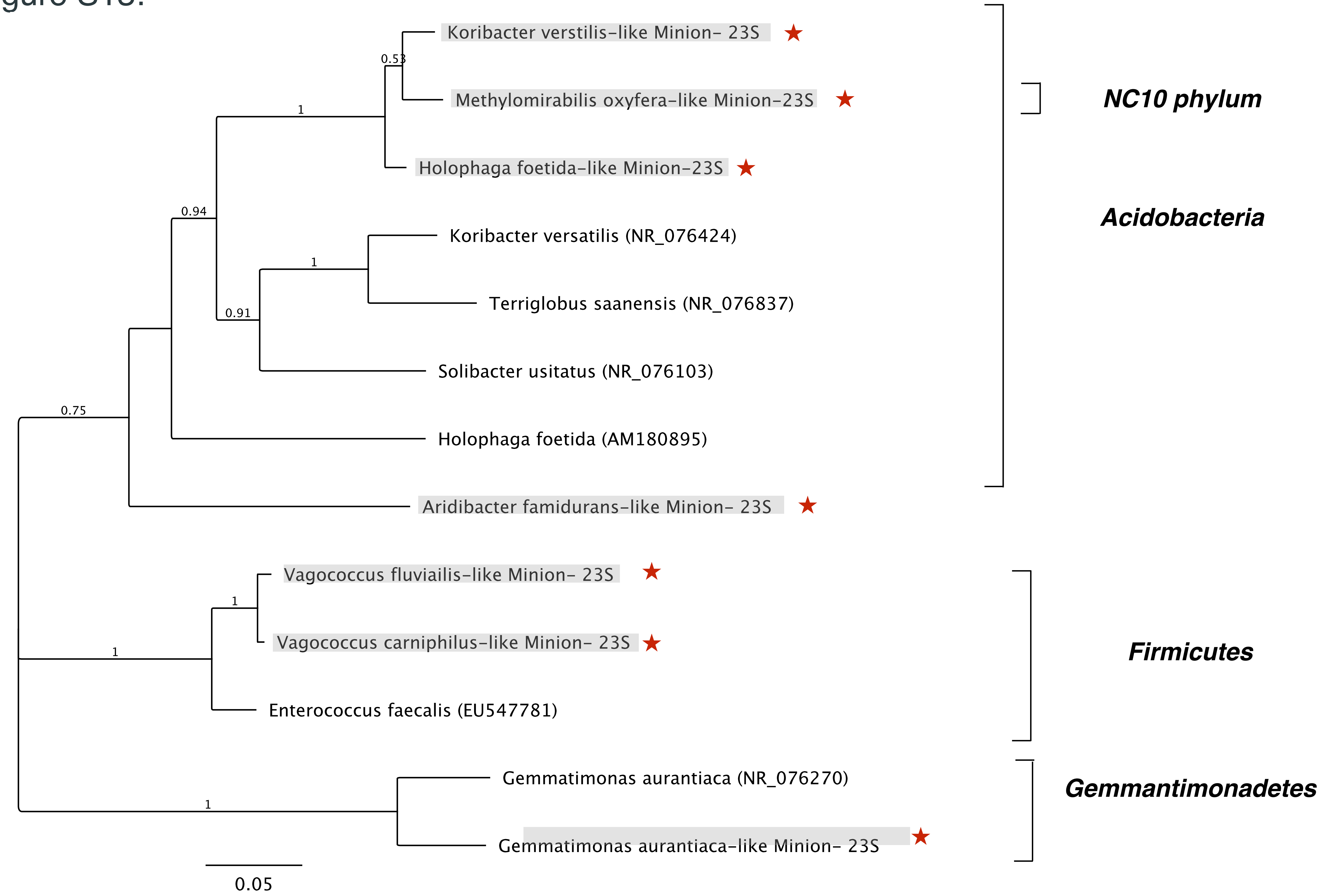


Tree reconstructions using FastTree for 1518 unambiguously aligned bases.

The MinION sequences are indicated by boxes and stars.

# Figure S14:

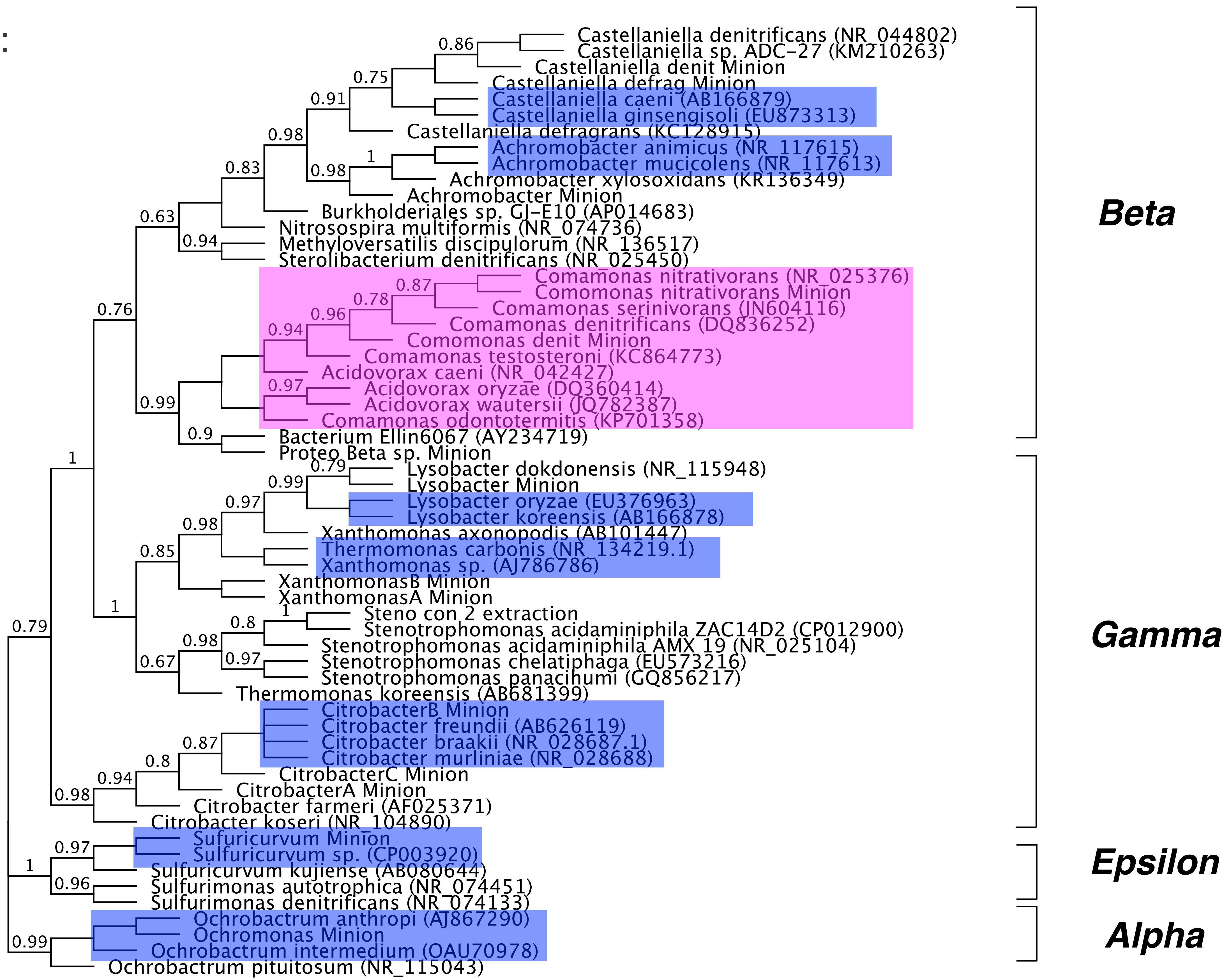


The portion of the tree not supported by bootstrap analysis is highlighted in blue while the portion of the tree not resolving bacterial genera is highlighted in pink.

Figure S15:

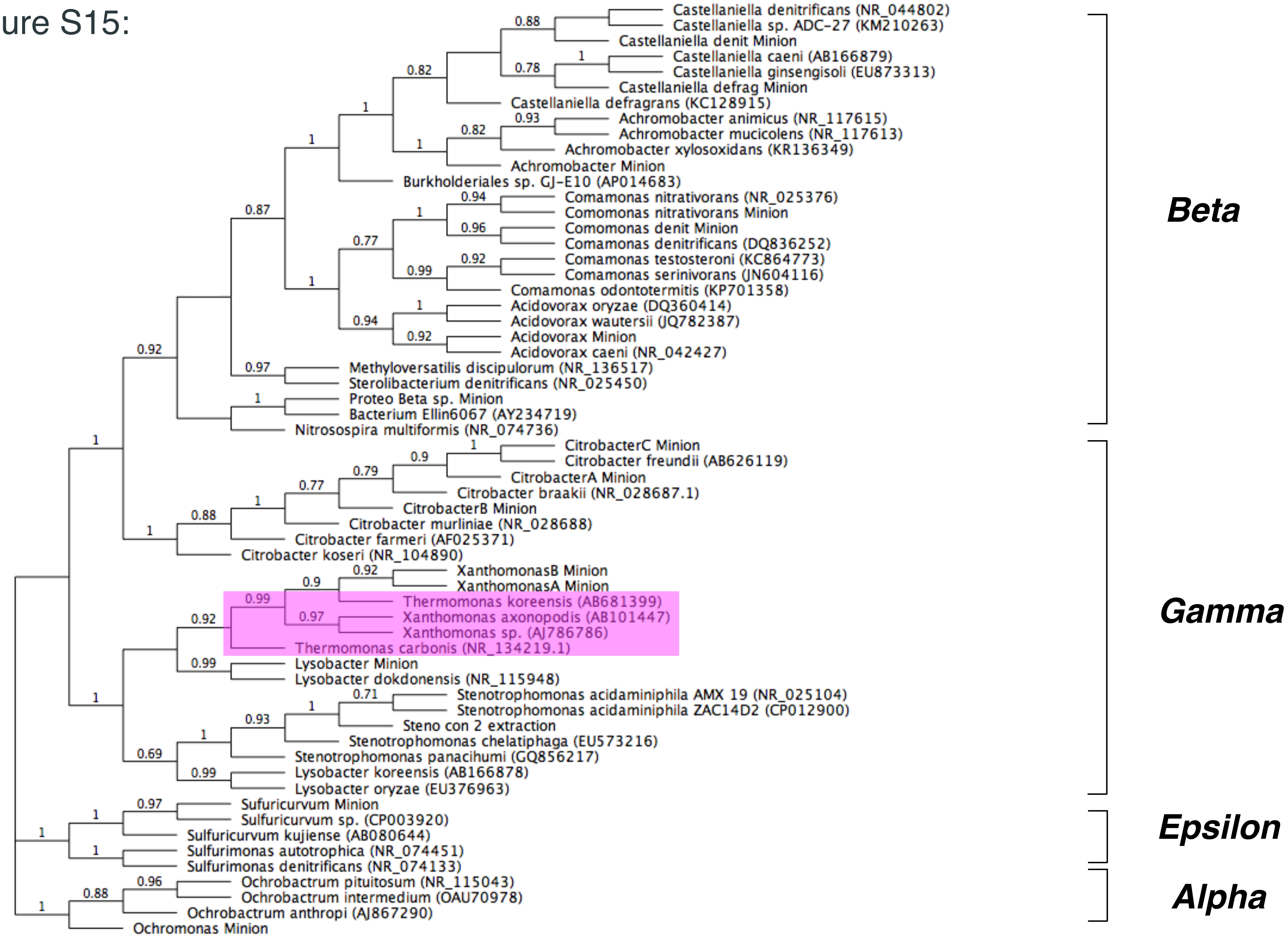


The portion of the tree not resolving bacterial genera is highlighted in pink.

Figure S16:

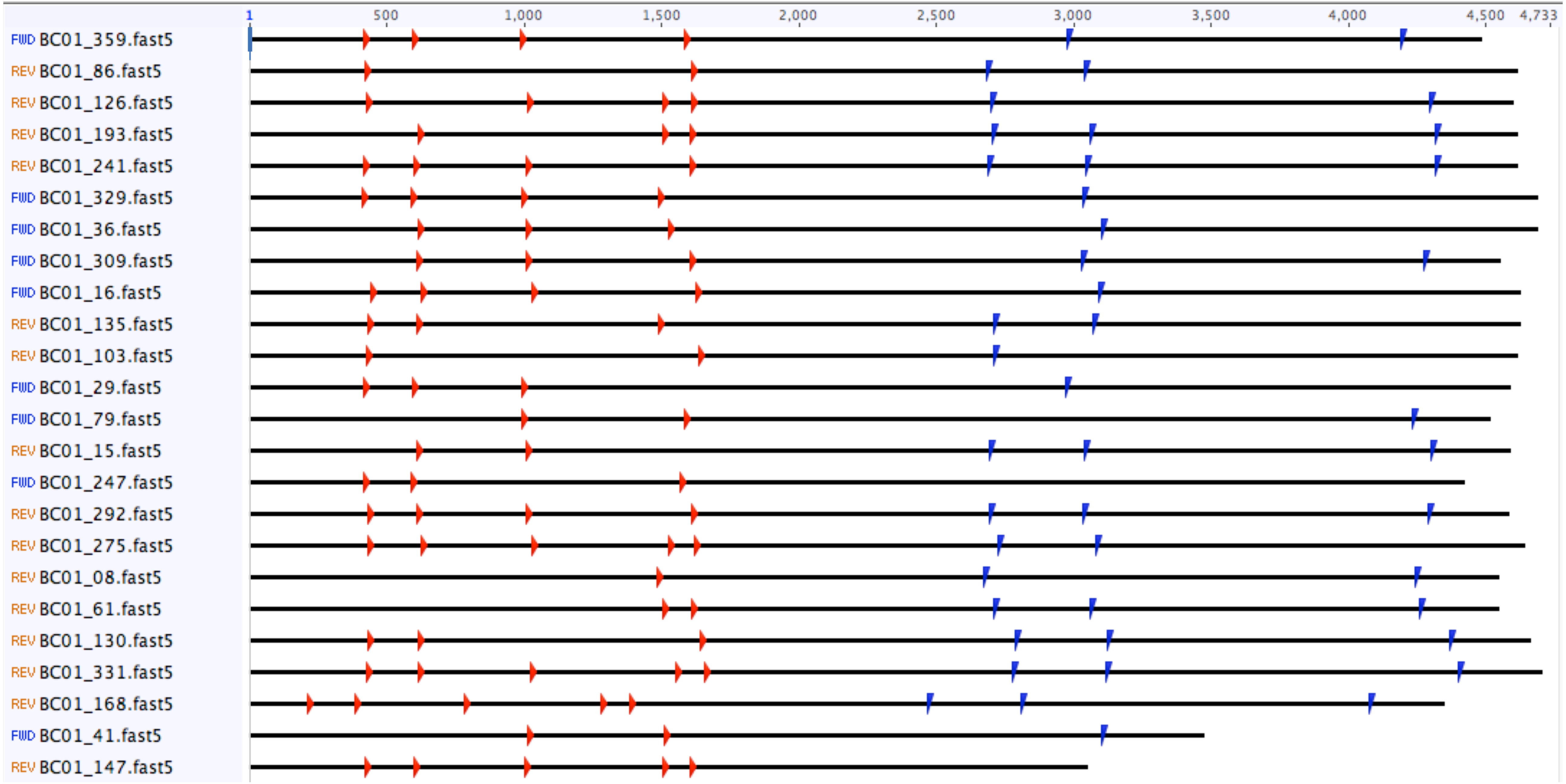


Universal 16S (red) and 23S (blue) rRNA gene priming sets are indicated.

Short sequences (<4000 bp) and those without priming sites are removed during QA/QC.

# Table S1:

| | 2D | 1D | Totals |
|---|---|---|---|
| **Total Reads** | 32991 | 56866 | 8.99E+04 |
| **Total bp** | 140533277 | 212346402 | 3.53E+08 |
| **Mean** | 4238 | 3698 | |
| **Median** | 4393 | 4090 | |
| **Min** | 828 | 13 | |
| **Max** | 8478 | 169741 | |
| **N25** | 4622 | 4767 | |
| **N50** | 4433 | 4383 | |
| **N75** | 4222 | 3811 | |

# Table S2

| Query | Description | Name | Grade | Query coverage | % Pairwise Identity |
|---|---|---|---|---|---|
| Sm100 | NR_041577.1 Stenotrophomonas maltophilia strain IAM 12423 16S ribosomal RNA gene, complete sequence | 9773 | 100% | 100% | 100% |
| Sm99 | NR_041577.1 Stenotrophomonas maltophilia strain IAM 12423 16S ribosomal RNA gene, complete sequence | 9773 | 99.5% | 100% | 98.9% |
| Sm98 | NR_041577.1 Stenotrophomonas maltophilia strain IAM 12423 16S ribosomal RNA gene, complete sequence | 9773 | 98.9% | 100% | 97.8% |
| Sm96 | NR_041577.1 Stenotrophomonas maltophilia strain IAM 12423 16S ribosomal RNA gene, complete sequence | 9773 | 97.9% | 100% | 95.8% |
| Sm94 | NR_041577.1 Stenotrophomonas maltophilia strain IAM 12423 16S ribosomal RNA gene, complete sequence | 9773 | 96.8% | 100% | 93.6% |
| Sm93 | NR_041577.1 Stenotrophomonas maltophilia strain IAM 12423 16S ribosomal RNA gene, complete sequence | 9773 | 96.4% | 100% | 92.8% |
| Sm91 | NR_041577.1 Stenotrophomonas maltophilia strain IAM 12423 16S ribosomal RNA gene, complete sequence | 9773 | 95.7% | 100% | 91.4% |
| Sm90 | NR_041577.1 Stenotrophomonas maltophilia strain IAM 12423 16S ribosomal RNA gene, complete sequence | 9773 | 95% | 100% | 89.9% |
| Sm89 | NR_041577.1 Stenotrophomonas maltophilia strain IAM 12423 16S ribosomal RNA gene, complete sequence | 9773 | 94.5% | 100% | 88.9% |
| Sm88 | NR_041577.1 Stenotrophomonas maltophilia strain IAM 12423 16S ribosomal RNA gene, complete sequence | 9773 | 94.1% | 100% | 88.1% |
| Sm87 | NR_041577.1 Stenotrophomonas maltophilia strain IAM 12423 16S ribosomal RNA gene, complete sequence | 9773 | 93.5% | 100% | 87% |
| Sm86 | NR_041577.1 Stenotrophomonas maltophilia strain IAM 12423 16S ribosomal RNA gene, complete sequence | 9773 | 92.8% | 100% | 85.5% |
| SM84 | NR_041577.1 Stenotrophomonas maltophilia strain IAM 12423 16S ribosomal RNA gene, complete sequence | 9773 | 92.1% | 100% | 84.1% |
| SM83Indel1 | NR_041577.1 Stenotrophomonas maltophilia strain IAM 12423 16S ribosomal RNA gene, complete sequence | 9773 | 91.6% | 100% | 83.1% |
| SM81Indel2 | NR_041577.1 Stenotrophomonas maltophilia strain IAM 12423 16S ribosomal RNA gene, complete sequence | 9773 | 90.7% | 100% | 81.4% |
| SM79Indel3 | NR_041577.1 Stenotrophomonas maltophilia strain IAM 12423 16S ribosomal RNA gene, complete sequence | 9773 | 90% | 100% | 79.9% |
| Cnitrati100 | NR_025376.1 Comamonas nitrativorans strain 23310 16S ribosomal RNA gene, partial sequence | 11440 | 100% | 100% | 100% |
| Cnitrarti99 | NR_025376.1 Comamonas nitrativorans strain 23310 16S ribosomal RNA gene, partial sequence | 11440 | 99.4% | 100% | 98.9% |
| Cnitrati98 | NR_025376.1 Comamonas nitrativorans strain 23310 16S ribosomal RNA gene, partial sequence | 11440 | 99% | 100% | 97.9% |
| Cnitrati96 | NR_025376.1 Comamonas nitrativorans strain 23310 16S ribosomal RNA gene, partial sequence | 11440 | 97.8% | 100% | 95.7% |
| Cnitrati95 | NR_025376.1 Comamonas nitrativorans strain 23310 16S ribosomal RNA gene, partial sequence | 11440 | 97.3% | 100% | 94.7% |
| Cnitrati93 | NR_025376.1 Comamonas nitrativorans strain 23310 16S ribosomal RNA gene, partial sequence | 11440 | 96.7% | 100% | 93.3% |
| cNitrati92 | NR_025376.1 Comamonas nitrativorans strain 23310 16S ribosomal RNA gene, partial sequence | 11440 | 95.8% | 100% | 91.7% |
| Cnitrati91 | NR_025376.1 Comamonas nitrativorans strain 23310 16S ribosomal RNA gene, partial sequence | 11440 | 95.4% | 100% | 90.9% |
| Cnitrati90 | NR_025376.1 Comamonas nitrativorans strain 23310 16S ribosomal RNA gene, partial sequence | 11440 | 95% | 100% | 90.1% |
| Cnitrati89 | NR_025376.1 Comamonas nitrativorans strain 23310 16S ribosomal RNA gene, partial sequence | 11440 | 94.4% | 100% | 88.9% |
| Cnitriti87 | NR_025376.1 Comamonas nitrativorans strain 23310 16S ribosomal RNA gene, partial sequence | 11440 | 93.5% | 100% | 87% |
| Cnitrati85 | NR_025376.1 Comamonas nitrativorans strain 23310 16S ribosomal RNA gene, partial sequence | 11440 | 92.7% | 99.93% | 85.4% |
| Cnitrati81Indel1 | NR_025376.1 Comamonas nitrativorans strain 23310 16S ribosomal RNA gene, partial sequence | 11440 | 91.3% | 99.94% | 82.7% |
| Cnitrati79Indel2 | NR_025376.1 Comamonas nitrativorans strain 23310 16S ribosomal RNA gene, partial sequence | 11440 | 90% | 99.94% | 80.1% |
| Cdenit100 | NR_025080.1 Comamonas denitrificans strain 123 16S ribosomal RNA gene, partial sequence | 11567 | 97.4% | 94.87% | 99.9% |
| Cdenit99 | NR_025080.1 Comamonas denitrificans strain 123 16S ribosomal RNA gene, partial sequence | 11567 | 96.7% | 94.87% | 98.6% |
| Cdenit97 | NR_025080.1 Comamonas denitrificans strain 123 16S ribosomal RNA gene, partial sequence | 11567 | 96% | 94.87% | 97.2% |
| Cdenti96 | NR_025080.1 Comamonas denitrificans strain 123 16S ribosomal RNA gene, partial sequence | 11567 | 95.3% | 94.87% | 95.7% |
| Cdenit95 | NR_025080.1 Comamonas denitrificans strain 123 16S ribosomal RNA gene, partial sequence | 11567 | 94.9% | 94.87% | 95% |
| Cdenit94 | NR_025080.1 Comamonas denitrificans strain 123 16S ribosomal RNA gene, partial sequence | 11567 | 94.3% | 94.74% | 93.9% |
| Cdenit92 | NR_025080.1 Comamonas denitrificans strain 123 16S ribosomal RNA gene, partial sequence | 11567 | 93.6% | 94.74% | 92.4% |
| Cdenit90 | NR_025080.1 Comamonas denitrificans strain 123 16S ribosomal RNA gene, partial sequence | 11567 | 92.4% | 94.74% | 90.1% |
| Cdenit89 | NR_025080.1 Comamonas denitrificans strain 123 16S ribosomal RNA gene, partial sequence | 11567 | 91.9% | 94.74% | 89% |
| Cdenit87 | NR_025080.1 Comamonas denitrificans strain 123 16S ribosomal RNA gene, partial sequence | 11567 | 90.9% | 94.74% | 87.2% |
| Cdenit86 | NR_025080.1 Comamonas denitrificans strain 123 16S ribosomal RNA gene, partial sequence | 11567 | 90.5% | 94.74% | 86.2% |
| Cdenit85 | NR_025080.1 Comamonas denitrificans strain 123 16S ribosomal RNA gene, partial sequence | 11567 | 89.6% | 94.74% | 84.4% |
| Cdenit81Indel1 | NR_025080.1 Comamonas denitrificans strain 123 16S ribosomal RNA gene, partial sequence | 11567 | 87.9% | 94.92% | 80.9% |
| Cdenti79Indel2 | NR_025080.1 Comamonas denitrificans strain 123 16S ribosomal RNA gene, partial sequence | 11567 | 87.1% | 94.89% | 79.3% |
